# Differential expression analysis of RNA-seq data at single-base resolution

**DOI:** 10.1093/biostatistics/kxt053

**Published:** 2014-01-06

**Authors:** Alyssa C. Frazee, Sarven Sabunciyan, Kasper D. Hansen, Rafael A. Irizarry, Jeffrey T. Leek

**Affiliations:** Department of Biostatistics, The Johns Hopkins University Bloomberg School of Public Health, 615 North Wolfe Street, Baltimore, MD 21205, USA; Department of Pediatrics, The Johns Hopkins University School of Medicine, 600 North Wolfe Street, Baltimore, MD 21287, USA; Department of Biostatistics, The Johns Hopkins University Bloomberg School of Public Health, 615 North Wolfe Street, Baltimore, MD 21205, USA

**Keywords:** Bioinformatics, Differential expression, False discovery rate, Genomics, RNA sequencing

## Abstract

RNA-sequencing (RNA-seq) is a flexible technology for measuring genome-wide expression that is rapidly replacing microarrays as costs become comparable. Current differential expression analysis methods for RNA-seq data fall into two broad classes: (1) methods that quantify expression within the boundaries of genes previously published in databases and (2) methods that attempt to reconstruct full length RNA transcripts. The first class cannot discover differential expression outside of previously known genes. While the second approach does possess discovery capabilities, statistical analysis of differential expression is complicated by the ambiguity and variability incurred while assembling transcripts and estimating their abundances. Here, we propose a novel method that first identifies differentially expressed regions (DERs) of interest by assessing differential expression at each base of the genome. The method then segments the genome into regions comprised of bases showing similar differential expression signal, and then assigns a measure of statistical significance to each region. Optionally, DERs can be annotated using a reference database of genomic features. We compare our approach with leading competitors from both current classes of differential expression methods and highlight the strengths and weaknesses of each. A software implementation of our method is available on github (https://github.com/alyssafrazee/derfinder).

## 1. Introduction

Microarrays revolutionized the way we measure gene expression by providing, for the first time, genome-wide transcript-level measurements, where *transcript* is used here to refer to the molecule associated with expression at the RNA level. However, assigning only one measurement to each known gene has greatly over-simplified the biological process in two ways. The first is that we have not yet discovered or annotated all regions of the genome capable of expressing transcripts. Secondly, most genes produce not one but several transcripts through the process of *alternative splicing* (Mortazavi *and others*, 2008; Trapnell *and others*, 2010; Katz *and others*, 2010). In principle, RNA-sequencing (RNA-seq) provides measurements of transcript expression from which we can obtain a more complete picture of reality. While microarrays rely on hybridization to predefined probes, by explicitly sequencing transcripts, RNA-seq is potentially capable of measuring expression in regions not previously annotated (Guttman *and others*, 2010; Clark *and others*, 2011), and to measure multiple transcripts for individual genes (Trapnell *and others*, 2010; Mortazavi *and others*, 2008). This flexibility, coupled with rapidly declining sequencing costs, has led to explosive growth in the use of RNA-seq technology (Stein and others, 2010).

The most common goal among investigators using either microarrays or RNA-seq is detecting differential expression, for example: discovering transcripts showing different average expression levels across two populations. A major difference between the two technologies is that in microarrays, measurement units are fixed in advance: only the abundances of the specific RNA sequences that correspond to probes on the microarrays are measured. With this approach, differential expression is relatively straightforward to quantify: measurements from the same probe are compared across samples. In contrast, RNA-seq reads out short sequences of molecules produced by shearing and reading RNA transcripts (the measurements produced are referred to as *reads*). Unlike with a microarray, across-sample comparisons are not straightforward as measurement units are not defined in advance. Therefore, reads must be summarized into units of expression before differential expression analysis can be performed. Different summarization approaches can lead to very different statistical inference.

Here, we group the most popular differential expression analysis approaches into two categories based on the ways that the reads are summarized. We refer to these two categories as (1) *annotate-then-identify* and (2) *assemble-then-identify*. The first category counts the number of reads that fall within previously identified boundaries of known genes. The second class seeks to assemble full transcripts directly from the reads. In either case, differential expression analysis is then performed on the resulting measurements at the gene or transcript level. In Section 2, we describe the limitations with the existing approaches and propose a new intermediate class of differential expression methods which we refer to as *identify-then-annotate*. In Section 3, we propose a specific implementation of the identify-then-annotate class of methods, which we call Differentially Expressed Region Finder (DER Finder). And in Section 4, using an example dataset, we show that identify-then-annotate models provide a good compromise between current RNA-seq analysis methods.

## 2. Differential expression analysis review

In this section, we review existing approaches to differential expression analysis with RNA-seq data and discuss how the philosophy behind DER Finder fits into this context. RNA-seq generates millions or billions of short sequences from individual mRNA molecules. Analyzing these sequence reads requires several steps: First, each read must be matched to the position it originates from in the genome in a process called alignment. Then, the number of reads aligned to specific regions must be summarized into quantitative measurements. The measurements are then normalized for the total number of reads measured for a particular sample and statistical models are applied to the summarized units. Oshlack and others (2010) describe this RNA-seq data analysis process in much more detail. Based on the summarization step, current statistical methods for the analysis of RNA-seq data can be grouped into two major classes. The methods in the first class, which we call annotate-then-identify, summarize the reads by counting the number that fall within pre-specified exons or genes. The exon and gene specifications, collectively called the *annotation*, are obtained from databases of previously identified genomic features.

Once the reads have been summarized at the exon or gene level, the statistical problem is very similar to statistical analysis of microarray data, with some deviations because the raw measurements take the form of counts. Note that the results from this step can be naturally summarized into matrices like those produced by microarray experiments, where rows are genes or exons and columns are samples. Therefore, many of the earliest statistical methods for analysis of RNA-seq data fall into this category because they were natural extensions of methods developed for microarrays. Two of the most widely used annotate-then-identify methods are EdgeR (Robinson *and others*, 2010; McCarthy *and others*, 2012) and DESeq (Anders and Huber, 2010); Alexa-seq (Griffith *and others*, 2010), DEXSeq (Anders *and others*, 2012), and a method developed by Wang and others (2012) are further examples of annotate-then-identify pipelines focusing on differential expression analysis of genomic structures that may indicate splicing or transcriptional differences between groups.

**Fig. 1.**
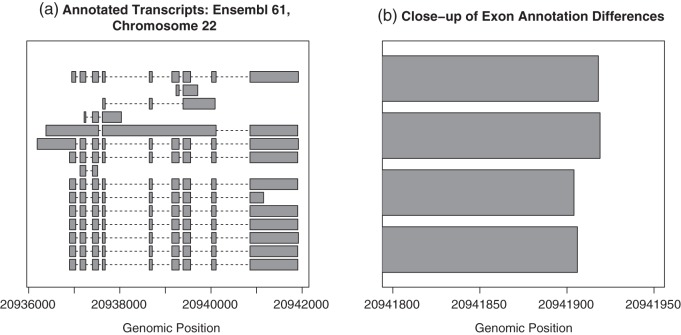
(a) Structures of annotated transcripts in a 6 kb region of the human genome (corresponding gene ID: ENSG00000099917). A transcript structure this complex causes problems in annotate-then-identify pipelines, as there is no clear way to determine which transcript or exon generated each read, especially if there is a high degree of overlap between unique features, as shown in (b): here, we zoom in on the exon on the right-hand side of (a) and see four overlapping yet distinct regions. Biologically, this could indicate a single exon with a varying transcription end site, but analytically, it introduces four potential counting regions and requires a critical counting decision to be made. Using a method like DER Finder eliminates the need for these decisions: if just one transcript or one form of an exon is differentially expressed, the genomic regions that uniquely identify that transcript or exon form will be called differentially expressed, and further analysis can be done on the small region to determine the exact phenomenon causing the observed pattern.

The annotate-then-identify approach provides a straightforward and interpretable analysis and that tested statistical methodology is available once raw read counts have been summarized into a gene-level matrix. However, one disadvantage is that it relies heavily on the accuracy of annotation databases of gene and exon boundaries, and current annotation may be unreliable or hard to interpret (Klimke and others, 2011). As shown in Fig. 1(a), the annotated transcript structure at individual genomic loci can be complex. Biologically, the distinct but overlapping regions in vertical columns represent a single exon used slightly differently in multiple transcripts. This complexity requires the analyst to make important counting decisions in advance, since each distinct use of an exon (represented by a box in Fig. 1(b)) represents a distinct potential counting region for annotate-then-identify methods. It is well known that different choices in how to count (all regions, only non-overlapping regions, or other choices) may lead to dramatically different results (Oshlack *and others*, 2010; Bullard *and others*, 2010), especially for genes whose transcripts have a low degree of similarity. In the case shown in Fig. 1, using a union model might allow for discovery of whole-gene differential expression, but it may mask a differential expression signal if, say, just one of the transcripts is overexpressed. Also, there is no “correct” gene model to use, so methods requiring this choice are at a disadvantage to those that do not. DER Finder does not require a gene model: if just a few transcripts or exons are differentially expressed, even in a complex scenario like Fig. 1 shows, the gene will simply be flagged as displaying a complicated differential expression pattern. This type of result is not possible in a gene-model-based approach. A second disadvantage of annotate-then-identify methods is that they do not allow for discovery of novel or previously uncharacterized exons or genes, since they rely on previously constructed databases.

The methods in the second class, which we call assemble-then-identify, attempt to assemble the full sequences of the mRNA molecules from which the short reads originated. These methods rely less heavily on annotation databases of exon or gene boundaries. Another advantage is that assemble-then-identify methods aim to fully quantify all the potential isoforms of mRNA molecules emanating from each gene. However, the short length of typical sequencing reads leads to inevitable ambiguity when attempting to assemble and quantify abundances of individual mRNA molecules: it is virtually impossible to determine which of many possible sets of assembled transcripts truly generated the observed RNA-seq data. This ambiguity also leads to varying and structured covariances between transcript measurements within genes, which complicates statistical analysis. There is also an increased computational cost associated with assembling full transcripts, quantifying their abundances, and performing transcript-level statistical tests, when compared with the more direct annotate-then-identify approach. The most widely used algorithm in this category is Cufflinks/Cuffdiff (Trapnell *and others*, 2010, 2012); others include Scripture (Guttman *and others*, 2010), and IsoLasso (Li and others, 2011). In our experience, the computational cost of transcriptome assembly is non-trivial: running Cufflinks (the transcript assembly step) took approximately 5 h on 4 standard cores for each sample, running Cuffmerge (merging 15 assemblies in preparation for DE analysis) took 1 h 39 min on 4 standard cores, and running Cuffdiff (assigning reads to transcripts and identifying DE) took about 42 h on 4 standard cores, comparing a 9-sample group with a 6-sample group. For comparison, alignment with Tophat took about 30 h per sample on 4 standard cores. Other researchers (Patro and others, 2013) have confirmed that assembly with tools other than Cufflinks also took several hours, and Cufflinks is one of the fastest assembly algorithms. Many of these tools allow the user to avoid the assembly problem by testing known transcripts for differential expression, but they then suffer from the previously mentioned shortcomings of annotate-then-identify methods.

Here, we propose an intermediate class of methods which we call identify-then-annotate. These methods first summarize the reads by counting the number of reads with alignments overlapping each individual base in the genome. Then we form a base-by-base statistic to identify bases that are differentially expressed between groups. Consecutive bases showing a common differential expression signature are grouped into DERs. The unit of statistical analysis is then the DER, which can be evaluated for statistical significance using permutation or bootstrap approaches. DERs can then be compared with previous databases of exons and genes to identify: (1) regions of differential expression corresponding to known exons or genes and (2) novel regions of differential expression. Currently, the closest analysis framework to an identify-then-annotate method is to combine pipelines: use an existing tool (e.g. rnaSeqMap Leśniewska and Okoniewski, 2011 or an assembler like Cufflinks) to identify expressed genomic regions, then test those regions for differential expression using existing statistical methods (e.g. Anders and Huber, 2010). Another identify-then-annotate pipeline has been proposed in the form of maximum mean discrepancy (Stegle *and others*, 2010) but does not have a software implementation available and is designed to test known genes for differential transcript expression—not to be run on an entire genome. We propose a new identify-then-annotate model that builds on the ideas behind the combining-pipelines approach: we feature a full statistical framework for expression detection and differential expression analysis.

The proposed identify-then-annotate model (1) allows for detection of differential expression in regions outside of known exons or genes, (2) allows for direct evaluation of differential expression of known genes and exons, (3) does not incur the added ambiguity and computational cost of assembly from short reads, and (4) can nonetheless detect differential splicing patterns and other expression differences between populations. Also, an identify-then-annotate tool can be used to address several commonly posed research questions at once, including differential expression, splicing analysis, and detection of novel features. For example, we could analyze differential expression of known features with annotate-then-identify tools, then use an assembly tool to detect novel features, then re-run the annotate-then-identify tool to analyze differential expression of the novel features—but an identify-then-annotate tool would address all of these issues at once. The primary disadvantage is that the proposed class of methods does not allow for direct quantification of alternative transcription. However, regions of potential alternative transcription can be easily identified where a subset of exons for a gene overlaps DERs but another subset does not, and those regions could be explored further with other tools.

## 3. DER Finder methodology

### 3.1 Base-level statistics

The first step in DER Finder is quantifying the evidence for differential expression at the nucleotide level. Since RNA-seq produces reads from mRNA transcripts, rather than directly from the genome, reads must be aligned using a strategy that accounts for reads that span intron-exon boundaries, called *junction reads*. In identify-then-annotate approaches like DER Finder, these junction reads are treated identically to reads that map directly to the genome when computing coverage. Tophat (Trapnell and others, 2009) is an example of an aligner that appropriately handles junction reads. The user must make choices about mapping parameters to use during the alignment step: for example, some reads will map to more than one genomic location (due to, e.g. repetitive regions or pseudogenes). Non-unique read alignments can either be discarded, in which case repetitive regions would not appear to be expressed at all, or kept, which would allow all repetitive regions to appear expressed but would not allow those regions to be distinguished from each other. Whatever alignment strategy and corresponding parameters are used, the result is ultimately a large matrix with rows corresponding to bases and columns corresponding to samples; entries of this matrix are the number of aligned reads from a particular sample that overlap a particular nucleotide. We refer to this matrix as the *coverage matrix*.

To quantify differential expression while accounting for biological variability and possible confounders, we fit a linear regression model to each row of the coverage matrix. Specifically, we let

(3.1) \begin{equation*}\label{eq3.1} g(Y_{ij}) = \alpha(l_j) + \sum_{p=2}^{P}\beta_{p}(l_j) X_{pi} + \sum_{k=1}^K \gamma_{k}(l_j)W_{ik} +\varepsilon_{ij}, \end{equation*}

where $Y_{ij}$ is coverage for sample $i$ at location $l_j$, $g$ is a Box–Cox style transformation (e.g. a log transformation) that makes the linear assumption acceptable, $\alpha (l_j)$ represents the baseline gene expression (coverage) level at location $l_j$, $X_{pi}$ is an indicator as to whether sample $i$ falls into category $p$ with category 1 being the reference group (e.g. when $P=2$, we have the case/control scenario, where $X_{2i}$ is a 0/1 indicator variable for whether sample $i$ is a case or a control), $\beta _p(l_j)$ is the parameter of interest quantifying differential expression between category $p$ and the reference category at location $l_j$ (e.g. in the case/control scenario, if $g$ is a log transform, then $\beta _2(l_j)$ represents the log fold change in expression for cases compared with controls), $W_{i k}$ ($k=1,\ldots ,K$) are the values of potential confounders for sample $i$, which may include sample-specific guanine/cytosine content effect (Hansen *and others*, 2012; Risso *and others*, 2011), sex or other demographic variables, or processing data, $\gamma _k(l_j)$ represents the effect of confounder $k$ on gene expression at location $l_j$, and $\varepsilon _{ij}$ represents residual measurement error at location $l_j$. Including confounders in this model is optional. We recommend setting $W_{i1}$ to be some measurement of library size for sample $i$ (e.g. median or 75th percentile of coverage for the sample across all bases).

Our goal is to segment the genome into contiguous regions $A$ where $\beta _p(l_j) \ne 0$ for at least one $p$ for all $l_j \in A$. Instead of modeling $\beta _p(l_j)$ as a functions (for example, with wavelet models or splines), we adopt a modular approach in which we first estimate $\beta _p(l_j)$ for each location $l_j$ and then divide the estimates into regions in a separate step. To estimate $\beta _p(l_j)$ along the genome and obtain test statistics from testing the null hypothesis that any of the $\beta _p(l_j)=0$, we can use methods for estimating regularized linear contrasts (Smyth *and others*, 2004), which take a shrinkage approach that is appropriate for small sample sizes and borrows information across bases. Details of this approach are available in supplementary material available at *Biostatistics* online.

### 3.2 Identifying candidate DERs with segmentation

In this section, we refer to the aforementioned test statistic resulting from the test for whether any $\beta _p(l_j)=0$ as $s(l_j)$. (For ease of notation, we omit the $j$ subscript in the discussion that follows). For most experiments, we expect the function $s(l)$ to be a step function that is mostly 0, since most of the genome is not differentially expressed. We do not expect $s(l)$ to be smooth because gene expression usually has a clear-cut start and end location. Hidden Markov models (HMMs) are a natural way of modeling $s$, and we describe the specifics of our implementation here.

We assume that there is an underlying Markov process along the genome $D(l)$ with three hidden states: $D(l) = 0$ if $\alpha (l)=\beta (l)=0$, $D(l) = 1$ if $\alpha (l)\neq 0$ and $\beta (l) = 0$, and $D(l) = 2$ if $\beta (l) \neq 0$. State $D(l) = 0$ corresponds to regions producing practically no gene expression. This state will be the most common, as most bases will not be covered by any reads because abundant gene expression is confined to a relatively small fraction of the genome. State $D(l)=1$ corresponds to regions for which gene expression is observed but does not differ between populations. We are interested in finding regions in the differentially expressed state, $D(l)=2$.

We assume that $D(l)$ is a first-order Markov chain with hidden state probabilities $\pi _d = {\mathrm {Pr}}(D(l) = d)$. We treat the transition matrix as fixed. As defaults, we set the retain state probabilities as very high with low transition probabilities between states, due to the sparsity of genes in the genome. The hidden state probabilities can be roughly estimated based on the relative frequencies of bases covered or not covered by genes, along with a prior estimate of the number of differentially expressed genes. DER Finder results are largely robust to changes in the prior estimates for $\pi _d$ (see Section 2.3 of supplementary material available at *Biostatistics* online).

Conditional on the hidden state of each base $l$, we then assume that $s(l)$ follows a normal distribution. Specifically, $s(l) \mid D(l) = d \sim N(\mu _d, \sigma _d^2)$. When $D(l) = 0$, there is little expression observed for base $l$, so we model the distribution as $N(0, \delta)$, where $\delta $ is an arbitrary, very small positive number, to restrict values to very close to zero. We estimate $\pi _0$ empirically by calculating the fraction of bases where the average coverage is less than a threshold $c$.

The model parameters for states $D(l) = 1$ and $D(l) = 2$ ($\mu _1$, $\mu _2$, $\sigma ^2_1$, and $\sigma ^2_2$) can be estimated using a standard two-groups mixture model, first proposed for the analysis of differential expression in microarray experiments (Efron, 2008). We assume that the statistics $s(l)$ from these two states are drawn from a mixture $f(s) = f_1(s)\pi _1^* + f_2(s)\pi _2^*$, where $\pi _1^* + \pi _2^* = 1$. (Estimates for $\pi _1^*$ and $\pi _2^*$ are scaled by the estimate of $\pi _0$ to obtain estimates for the overall state probabilities, $\pi _1$ and $\pi _2$, such that $\pi _0+\pi _1+\pi _2=1$.) Each mixture component is again assumed to be normal and can be estimated using the empirical null distribution. We can then directly estimate the most likely path of unobserved states $D(l)$ based on the observed statistics $s(l)$ using standard estimation techniques for HMMs. Details on the specific form of the test statistics, the parameters of the HMM, and validity of HMM assumptions are available in Sections 1–2 of supplementary material available at *Biostatistics* online.

### 3.3 Statistical significance

The HMM essentially segments the genome into regions, where a region is defined as a set of contiguous bases having the same predicted latent state. A region of bases with predicted latent state $D(l)=2$ is referred to as a candidate DER. Beyond the segmentation step in the DER Finder pipeline, all analysis is done on the region level rather than the base level. Region-level analysis ensures that the number of statistical tests is not unreasonably large (as it would be if we did a formal test at every base) and makes it such that variations in read coverage at individual bases that can arise due to technical artifacts in RNA-seq data will not affect the final results.

After segmenting the genome into regions, we assign a $p$-value to each candidate DER using a permutation procedure. In calculating the $p$-values for each candidate DER, we consider the size of the individual statistics within each region, since regions with very large test statistics are more likely to be truly differentially expressed. We apply an approach similar to Jaffe and others (2012): first, we calculate the average base-level test statistic within each potential DER $r:\bar {s}_r = \sum _{l \in {\rm DER} r} s(l)$. Note that $\bar {s}_r$ is the region-level test statistic for region $r$. In the simple case–control scenario with no confounders, we can assign $p$-values to DERs with the following permutation procedure:

1. Permute the values of the covariate of interest ($X_{i}$) for all samples.
2. Re-calculate the base-level statistics using (3.1). Denote these null statistics by $s^0(l)$.
3. Re-run the HMM on the $s^0(l)$s to identify a set of null DERs, indexed by $\rho $ and denoted by DER$^0_{\rho }$.
4. To form region-level null test statistics, calculate the average base-level statistic within each null DER $\bar {s}^0_\rho = \sum _{l \in {\mathrm {DER}}^0 \rho } s^0(l).$

Steps 1–4 are repeated $B$ times, and the empirical $p$-value for region $r$ is $p_r = ({1}/{\sum _{b=1}^B P_b})\sum _{b=1}^B \sum _{\rho =1}^{P_b}1(\bar {s}^0_{\rho } > \bar {s}_r)$, where $P_b$ is the number of null DERs for permutation $b$. This quantity is the percent of null DERs with average statistic as or more extreme than the observed statistic for candidate DER $r$ calculated on the observed data. Standard false discovery rate calculations can then be applied to adjust these $p$-values for multiple testing.

In the case where confounders or additional covariates are included in model (3.1), a straightforward bootstrap extension of this permutation approach can be derived. After assigning statistical significance to each region, the DERs can be annotated using a reference database of known genomic features; an example of an annotation procedure can be found in supplementary material available at *Biostatistics* online.

## 4. Results: comparison on real data

Our method is designed for differential expression with biological replicates, but many published experiments do not include such replicates (Hansen *and others* 2011). We therefore designed an analysis comparing brain tissue between nine human males and six human females to assess the competing methods: the Y chromosome was tested for differential expression between sexes using DER Finder, Edge R, and DESeq (using previously annotated exons) and Cufflinks/Cuffdiff. Specific details of the experiment can be found in Section 4 of supplementary material available at *Biostatistics* online.

Two sets of results were obtained: one analysis compared males to females, and the other compared a randomly selected set of five of the males to the other four males. We expect virtually all genomic features of the Y chromosome (barring the pseudoautosomal region, pseudogenes, and other irregularities) to be differentially expressed between males and females, since females do not have a Y chromosome, and no genomic features to be differentially expressed between control males.

### 4.1 DER Finder results

DER Finder identified 534 Y-chromosome regions as differentially expressed ($q<0.05$) between males and females. Six of these regions were classified as underexpressed in males, which we know to be an artifact, but the other 528 were identified as overexpressed in males as expected. Additionally, we found 333 novel differentially transcribed regions ($q < 0.05$). These novel transcribed regions ranged in length from 1 to 3814 bases. These regions may indicate noise from the method, but they also may point to regions that should be examined further, either because they have interesting mapability characteristics or because they might truly be expressed and not yet annotated. The 534 DERs pointed to 411 differentially expressed exons, using the criteria outlined in Table 1 of supplementary material available at *Biostatistics* online. These 411 exons came from 33 different genes, which means we found those 33 genes to be differentially expressed or indicate an event of interest. In comparing males with each other, we did not identify any differential expression on the Y chromosome: the minimum $q$-value for the regions found to be differentially expressed in the HMM step was 0.86.

### 4.2 Cufflinks/Cuffdiff results

Of 808 assembled transcripts tested for differential expression on the Y chromosome between males and females, the Tophat–Cufflinks–Cuffdiff pipeline found no differentially expressed transcripts. The minimum $q$-value for these assembled transcripts was 0.45. While 736 of these transcripts showed non-zero abundance in males and zero abundance in females, these differences were not found to be statistically significant using the Cuffdiff methodology. Similar, too-conservative results were reported in supplementary material available at *Biostatistics* online of the manuscript accompanying the release of Cuffdiff version 2 (Trapnell *and others* 2012). In the comparison of normal males, none of the 818 assembled transcripts were called differentially expressed: the minimum $q$-value was 0.63.

### 4.3 EdgeR and DESeq results

Both of these methods tested 433 exons on the Y chromosome for differential expression between males and females. The other annotated exons on the Y chromosome did not have any reads mapping to them or the counting model did not allow any reads to be counted for them. Of these 433 exons, EdgeR classified 113 and DESeq classified 115 as differentially expressed between males and females ($q<0.05$). Ninety-seven exons were found by both EdgeR and DESeq. When comparing the males with each other, neither method found any exons to be differentially expressed: all $q$-values were 1 except for 2 exons with $q = 0.12$ in EdgeR.

### 4.4 Comparison of results across methods

**Fig. 2.**
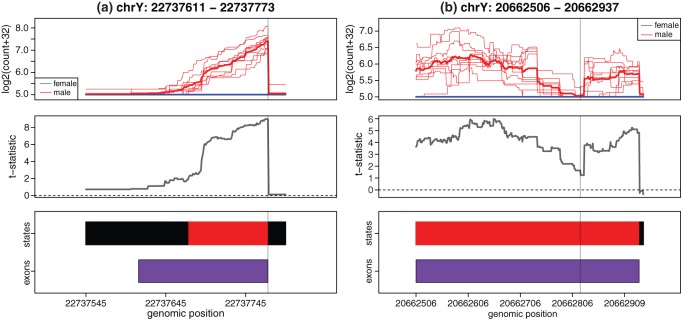
Cases where DER Finder correctly calls differential expression and annotate-then-identify methods do not. (a) Example of an exon (from gene EIF1AY, Ensembl exon id ENSE00001435537) whose location appears to be mis-annotated, leading EdgeR and DESeq to underestimate the exon's abundance and therefore incorrectly call this exon not differentially expressed. (b) Example of a DER ($q = 0.001$) falling outside of an annotated exon, which can be found by DER Finder but not by EdgeR or DESeq. Although there are no annotated exons in this region, we believe this finding is more than noise because it is supported by the following annotated ESTs: DR001278, BF693629, BF672674, BM683941, BM931807, and CD356860 (GenBank accession numbers. Top panels: single-base resolution coverage (on log2 scale). Middle panels: $t$-statistics from linear model fit by DER Finder. Bottom panels: exon locations and state calls from DER Finder: light gray $=$ not expressed, black $=$ equally expressed, red or dark gray $=$ overexpressed in men).

DER Finder exhibits performance comparable with that of EdgeR and DESeq, while all three methods outperform Cufflinks/Cuffdiff. DER Finder also has major advantages over EdgeR and DESeq: DER Finder is agnostic to annotation, which means it can identify differential expression signal in two important cases: (a) the case where a feature may be slightly mis-annotated or where the read mappings do not quite match up with the feature's annotation and (b) the case where differential expression exists in regions that do not overlap annotated features. Both these scenarios occurred in the dataset studied. Fig. 2(a) illustrates a case where the location or length of an exon may be incorrectly annotated. In that example, the mis-annotation caused the exon's expression to be underestimated when counting reads overlapping it. As a result, the statistical tests used in EdgeR and DESeq did not have enough power to call this Y-chromosome exon differentially expressed (both tools report $q = 1$) between males and females. DER finder more accurately reported the shown DER as overlapping 61.3% of an annotated exon with a $q$-value $=$ 0.001. Also, DER Finder can find regions of interest that fall outside of annotated exons (Fig. 2(b)). Closer inspection of the illustrated region using the UCSC Genome Browser (build: hg19, tracks: UCSC genes, RefSeq genes, Human ESTs, Spliced ESTs) reveals no annotated genes in the region, but shows that the expression is supported by six ESTs, providing evidence that the signal here is truly biological rather than simply background noise. Section 5 of supplementary material available at *Biostatistics* online discusses other advantages of DER Finder over identify-then-annotate methods. Additional analysis of the agreement between DER Finder's results and those of Cufflinks/Cuffdiff, EdgeR, and DESeq can also be found in Section 6 of supplementary material available at *Biostatistics* online. DER Finder's ability to find differential expression signal even in the presence of wrong or missing annotation is a key advantage over annotate-then-identify methods.

**Fig. 3.**
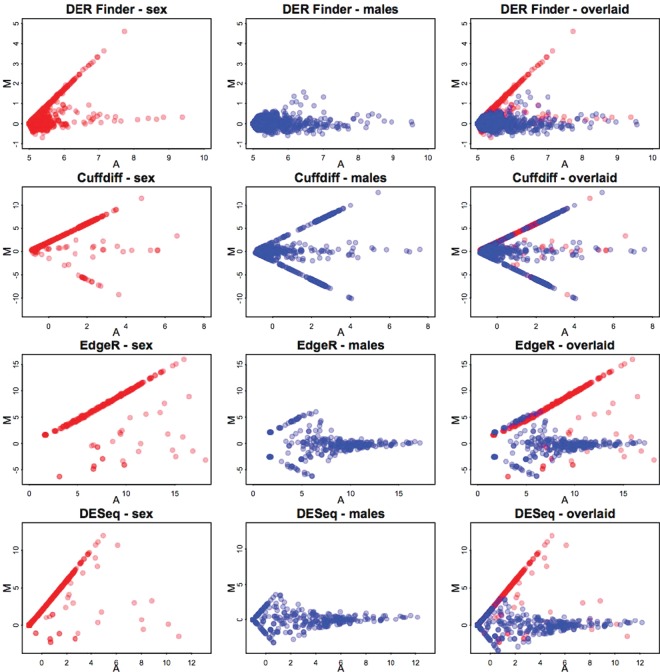
$MA$ plots for Y-chromosome regions, transcripts, or exons, for each method and for both male vs. female (red) and male vs. male (blue) comparisons. On each plot, the $x$-axis represents the average log (base 2) abundance for each unit (region for DER Finder, transcript for Cufflinks, exon for EdgeR and DESeq), and the $y$-axis represents the log (base 2) fold change between males and females (red points) or the two groups of males (blue points). We expect to see the red, positively sloped diagonal on all plots: this represents genomic regions expressed in males but not in females. In DER Finder, EdgeR, and DESeq, this diagonal corresponds with differential expression detected, however, no differential expression was detected in Cufflinks even though the red diagonal exists as expected. The displayed $M$ and $A$ values for EdgeR and DESeq are normalized. Specifically, the EdgeR plot is logCPM vs. logFC, where logCPM is $\log _2$ counts-per-million and logFC is the $\log _2$ fold change (male to female); both are normalized for library size and dispersion and are reported in the output of the exactTest function. The DESeq plot is log_2_(baseMeanA+0.5) + log_2_(baseMeanB + 0.5))/2 vs log_2_(baseMeanA+0.5) − log_2_(baseMeanB +0.5), where baseMeanA and baseMeanB represent library-size-normalized counts for males and females, respectively, and are reported in the output table from the function nbinomTest. Since baseMeanA and baseMeanB were sometimes 0, we added 0.5 as an offset to avoid calculating $\log _2(0)$.

To assess whether the tools give reasonable results and to compare overall performance of the three methods, $MA$ plots (Dudoit *and others* 2002) were used to show the relationship between each unit's average expression (denoted with $M$) and the magnitude of differential expression it exhibits (denoted with $A$). The unit is a region for DER Finder, an exon for EdgeR and DESeq, or a transcript for Cufflinks. The $MA$ plots resulting from the Y-chromosome experiment (Fig. 3) reveal that DER Finder, EdgeR, and DESeq all produce reasonable results, but the findings from Cufflinks are somewhat problematic. While there does seem to be more overexpression of transcripts in males in the male/female differential expression analysis done by Cufflinks, we observe several extreme fold changes in the opposite direction, and the male-to-male comparison also produced these extreme fold changes. These problems do not exist in the other methods, whose $MA$ plots illustrate high fold changes found between males and females and very little change found between males, as expected.

**Fig. 4.**
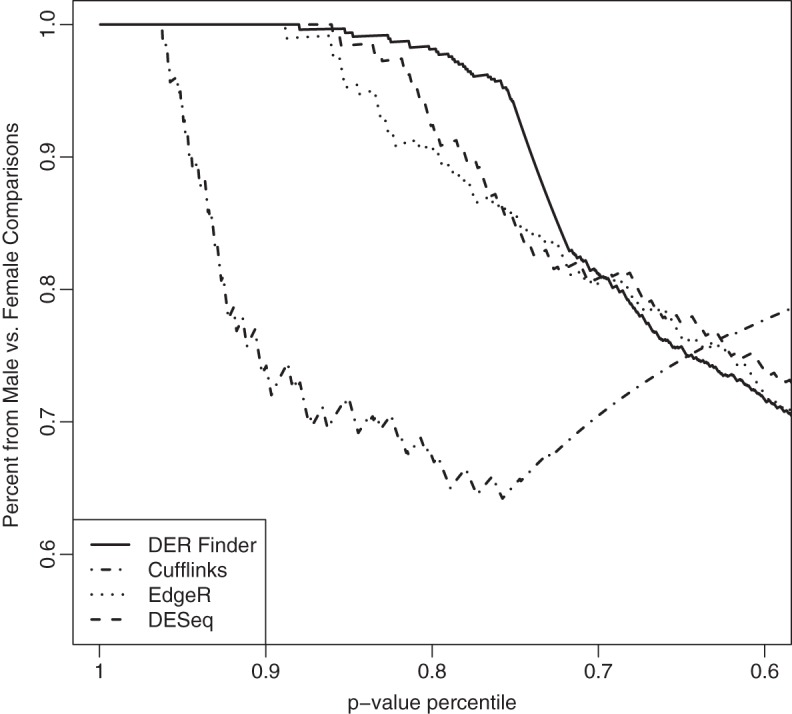
Percentage of significantly DERs/transcripts/exons originating from male-to-female comparisons, using various percentiles of the $p$-value distribution as a significance cutoff. We find that most highly significant results are true positives, i.e. results with low $p$-values and high test statistics stem from comparing males with females, for DER Finder, EdgeR, and DESeq, while Cufflinks exhibits problems in this area.

Finally, to get a sense of each method's accuracy, we evaluated the tables of DERs between sexes and between males produced by each method. We gathered all resulting regions—both negative results, from the male vs. male comparison, and positive results, from the male vs. female comparison—and ordered them by the value of their test statistic. An algorithm ranking all positive results ahead of the negative ones is preferred. Fig. 4 shows, at each percentile of the differential expression test statistic, the percent of regions that are positive. This is analogous to finding the percentage of findings that were truly positive at different significance cutoffs, assuming all tests in the sex comparison should be positives and tests in the male comparison should be negatives. We find that EdgeR, DESeq, and DER Finder perform comparably: all or most of the top 20% of regions, ranked by test statistic, came from comparisons between sexes. Cufflinks does much worse: only about 60% of the top 20% of their top transcripts came from the male-to-female comparison. DER Finder performs just slightly better than EdgeR and DESeq in addition to having other advantages over these methods, as discussed earlier.

## 5. Discussion

We propose DER Finder as a specific implementation of a new class of methods for differential expression analysis of RNA-seq data. The new class deals with identified challenges by (a) not relying on existing annotation when calling differential expression and (b) avoiding the immensely difficult problem of full transcript assembly by putting differential expression into a more straightforward framework. We have built on the ideas behind the approach of combining pipelines (e.g. rnaSeqMap combined with DESeq) to create a full pipeline for statistical analysis of differential expression. DER Finder outperforms Cufflinks/Cuffdiff and performs comparably with EdgeR and DESeq, while having the added advantages of sensitivity even in the presence of incorrect annotation and transcript discovery capability. We have also considered DER Finder's performance in other scenarios: a simulation study is presented in Section 7 of supplementary material available at *Biostatistics* online that addresses experimental design questions and examines DER Finder's accuracy. An identify-then-annotate method like DER Finder is an important step in developing new ways to analyze RNA-seq data, so further properties of these types of methods are worth investigating.

## 6. Software

All software and code used in this analysis is available on github (https://github.com/alyssafrazee/derfinder).

## Supplementary material

Supplementary material is available at http://biostatistics.oxfordjournals.org.

## Funding

R.A.I. was partially supported by NIH Ro1 HG005220. J.T.L. and K.D.H. were partially funded by NIH Ro1 GM105705-01. J.T.L. was partially funded by NIH P50 MH-094268 Silvo O. Conte Center. S.S. was provided by the Stanley Brain Research Institute. Funding to pay the Open Access publication charges for this article was provided by Jeffrey Leek's discretionary fund.

## Supplementary Material

Supplementary Data
