# Corrigendum: Differential expression analysis of RNA-seq data at single-base resolution

**DOI:** 10.1093/biostatistics/kxu022

**Published:** 2014-07

**Authors:** Alyssa C. Frazee, Kasper D. Hansen, Jeffrey T. Leek, Sarven Sabunciyan, Rafael A. Irizarry

**Affiliations:** Department of Biostatistics, The Johns Hopkins University Bloomberg School of Public Health, 615 North Wolfe Street, Baltimore, MD 21205, USA; Department of Pediatrics, The Johns Hopkins University School of Medicine, 600 North Wolfe Street, Baltimore, MD 21287, USA; Dana Farber Cancer Institute, 450 Brookline Avenue, Boston, MA 02215, USA

(10.1093/biostatistics/kxt053)

## 1. Statistical significance: updated definition of $\bar {s}$

Section 3.3 of the main manuscript details the permutation $p$-value procedure implemented in the DER Finder method. Incorrect definitions were given for $\bar {s}_r$ and $\bar {s}_{\rho }^0$. The correct definitions are:

\[ \bar{s}_r =\dfrac{\sum_{l \in DER_r} s(l)}{\text{length}(DER_r)}\hbox{ and } \bar{s}_{\rho}^0 = \dfrac{\sum_{l \in DER^0_{\rho}} s^0(l)}{\text{length}(DER^0_{\rho})}. \]

The original manuscript incorrectly presented these region-level test statistics as sums, when they should have been presented as averages. This correction does not affect the manuscript’s results: the correct region-level test statistics were used in the analysis; only the written definition of the region-level test statistics was incorrect.

## 2. Updated Figure 2(b)

An incorrect version of Figure 2(b) was presented in the original manuscript. The DER presented in Figure 2(b) actually does overlap an annotated exon (as demonstrated in the bottom panel of the original figure!), which contradicts the message of the figure caption. The corrected version of Figure 2 appears below.

**Figure 2.**
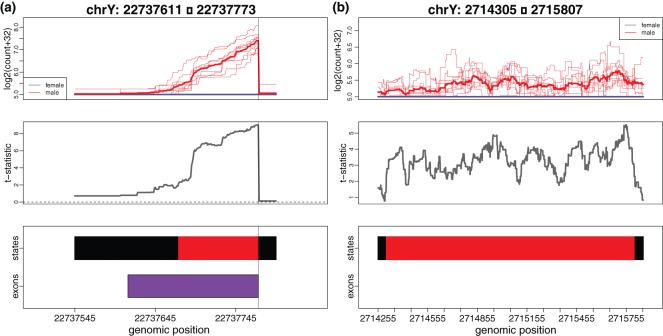
Cases where DER Finder correctly calls differential expression and annotate-then-identify methods do not. (a) Example of an exon (from gene EIF1AY , Ensembl exon id ENSE00001435537) whose location appears to be mis-annotated, leading EdgeR and DESeq to underestimate the exon’s abundance and therefore incorrectly call this exon not differentially expressed. (b) Example of a differentially expressed region ($q = 0.001$) falling outside of an annotated exon, which can be found by DER Finder but not by EdgeR or DESeq. Though there are no annotated exons in this region, we believe this finding is more than noise because it is supported by the following annotated ESTs: CT001420, BF810102, BF369919, BF858017, CV424981 (GenBank accession numbers). Top panels: single-base resolution coverage (on log2 scale). Middle panels: $t$-statistics from linear model fit by DER Finder. Bottom panels: exon locations (denoted by purple boxes) and state calls from DER Finder: gray $=$ not expressed, black $=$ equally expressed, red $=$ overexpressed in men. This figure appears in colour online.

## 3. Correction to Supplemental material

We note that there is also a published correction to the supplement that was originally published with this manuscript.

## Supplementary Material

Supplementary Data

