# Supplementary material for: Differential expression analysis of RNA-seq data at single-base resolution
Source: Biostatistics. 2014 Jan 6;15(3):413–26. doi: 10.1093/biostatistics/kxt053 (PMC4059460; doi:10.1093/biostatistics/kxt053)
Supplement: Supplementary Data [file supp_15_3_413__index.html]

Differential expression analysis of RNA-seq data at single-base resolution — Supplementary Data 

# Differential expression analysis of RNA-seq data at single-base resolution

## Supplementary Data

Supplementary Data

**Files in this Supplementary Material:**

- Supplementary Data - Pdf file
