# Supplementary material for: Differential expression analysis of RNA-seq data at single-base resolution
Source: Biostatistics. 2014 Jan 6;15(3):413–26. doi: 10.1093/biostatistics/kxt053 (PMC4059460; doi:10.1093/biostatistics/kxt053)
Supplement: Supplementary Data [file supp_kxt053_kxt053supp_data.pdf]

Biostatistics (2014), **0**, 0, *pp.* 1–31
doi:10.1093/biostatistics/correction˙B

Original and Corrected supplementary material to: Differential expression analysis of RNA-seq data at single-base resolution

Alyssa C. Frazee[1], Sarven Sabunciyan[2], Kasper D. Hansen[1], Rafael A. Irizarry[3], Jeffrey T. Leek[1*]

1. Department of Biostatistics, The Johns Hopkins University Bloomberg School of Public Health, 615 North Wolfe Street, Baltimore, MD 21205, USA

2. Department of Pediatrics, The Johns Hopkins University School of Medicine, 600 North Wolfe Street, Baltimore, MD 21287, USA

3. Dana Farber Cancer Institute, 450 Brookline Avenue, Boston, MA 02215, USA

jtleek@gmail.com

1. Details on Segmentation and Hidden Markov Model in *derfinder*

In *derfinder*, we fit linear models (as specified by equation (3.1) in the main text) at each base in the genome. To do this, we use methods for estimating regularized linear contrasts as implemented in the *limma* Bioconductor package (Smyth *and others* 2004, Smyth 2005). We use a customized version of the `lmFit` function, keeping the default parameters. For the two-group comparison presented in the manuscript, the test statistic $s(l)$ is a moderated t-statistic, which is similar to the ordinary t-statistic obtained from testing whether $\beta_2(l) = 0$, but the standard error estimate for $\beta_2(l)$ used it its calculation is shrunk toward a prior variance estimate. This framework allows for the borrowing of information across bases, which makes the statistical results more reliable

© The Author 2014. Published by Oxford University Press. All rights reserved. For permissions, please e-mail: journals.permissions@oup.com

in experiments with small sample sizes. To be more specific, we present some of the details from Smyth *and others* (2004) here; further details can be found in that paper. For ease of notation, since we are in two-group case, we drop the "2" subscript from $\beta_2(l)$ in the following discussion. Following the framework from Smyth *and others* (2004), we assume a distribution on the estimated differential expression effect at base l:

$$\hat{\beta(l)} \mid \beta(l), \sigma_l^2 \sim N(\beta(l), v_l\sigma_l^2)$$

where σ_l^2 represents the residual variance and v_l represents the unscaled variance at base l. We also assume a distribution on the estimated residual variance for the model at base l, assuming d_l is the residual degrees of freedom for that model:

$$s_l^2 \mid \sigma_l^2 \sim \frac{\sigma_l^2}{d_l}\chi^2_{d_l}$$

Then, a prior with parameters s_0^2 and d_0 is assumed on σ_l^2:

$$\frac{1}{\sigma_l^2} \sim \frac{1}{d_0 s_0^2}\chi^2_{d_0}$$

The prior describes how variances are expected to vary across bases. A prior is also assumed on $\beta(l)$ when $\beta(l) \neq 0$:

$$\beta(l) \mid \sigma_l^2 \sim N(0, v_{0l}\sigma_l^2)$$

This prior describes the distribution of differential expression parameters (here, log fold-changes) for differentially expressed bases. Under these priors, the posterior mean of σ_l^{-2} given s_l^2 is $\tilde{s}_l^{-2}$, where:

$$\tilde{s}_l^2 = \frac{d_0 s_0^2 + d_l s_l^2}{d_0 + d_l}$$

Our test statistic $s(l)$, here the moderated t-statistic at base l, is then defined by:

$$s(l) = \tilde{t}_l = \frac{\hat{\beta(l)}}{\tilde{s}_l\sqrt{v_l}}$$

This empirical Bayes approach, where the posterior variance is used in the t-statistic calulation instead of the sample variance, is implemented in the `eBayes` function in *limma*. Data-driven

estimation of the values of d_0 and s_0^2 is built into the `eBayes` function, as described in Section 6 of Smyth *and others* 2004. This function has been incorporated into *derfinder*'s functions.

The Hidden Markov Model is then fit on the moderated t-statistics. In the general case, described in the main text, we assume a three-state Markov process D along genomic locations l, such that $D(l) = 0$ when base l is not expressed, $D(l) = 1$ when base l is equally expressed between conditions, and $D(l) = 2$ when base l is differentially expressed. However, in our implementation, we found it convenient to divide the differentialy expressed state into two separate states. So in *derfinder*, we define $D(l) = 0$ and $D(l) = 1$ the same way we do in the general case, but we assume here that $D(l) = 2$ corresponds to overexpression of base l in cases (compared to controls) and $D(l) = 3$ corresponds to underexpression of base l in cases.

As input, the HMM requires several parameters: a transition matrix (defining probabilities of transitioning from one hidden state to another in consecutive base-pairs), fixed probabilities of being in each hidden state, and parameters defining the distribution of $s(l) \mid D(l)$. For transition probabilities, *derfinder* uses the following matrix as defaults:

$$\begin{bmatrix} 0.999 & (1/3) * 0.001 & (1/3) * 0.001 & (1/3) * 0.001 \\ 0.001 - 2 \times 10^{-12} & 0.999 & 1 \times 10^{-12} & 1 \times 10^{-12} \\ 0.001 - 2 \times 10^{-12} & 1 \times 10^{-12} & 0.999 & 1 \times 10^{-12} \\ 0.001 - 2 \times 10^{-12} & 1 \times 10^{-12} & 1 \times 10^{-12} & 0.999 \end{bmatrix} \tag{1.1}$$

where entry (k, k') $(k = 1, 2, 3, 4)$ of 1.1 defines $Pr(D(l) = k - 1 \mid D(l-1) = k' - 1)$. Low probabilities are intentionally assigned to transitions from a differentially expressed state to an equally expressed state and vice versa, based on the assumption that discrete genomic features are not usually only partially differentially expressed. These parameters may be changed by the user in the *derfinder* package. Initial tests of *derfinder* indicate that the method is not sensitive to changes in the parameters of the transition matrix as long as the diagonal entries are large probabilities.

The parameters left to estimate are $\pi_d = Pr(D(l) = d)$, μ_d, and σ_d^2 for $d = 0, 1, 2, 3$. Recall that we assume $s(l) \mid D(l) = d \sim N(\mu_d, \sigma_d^2)$. We estimate π_0 as the fraction of bases where

average coverage is less than some threshold c, as described in the text. Estimates of π_1, π_2, and π_3 are obtained from the maximum likelihood approach of the two-groups model (Efron 2008). This model estimates π_1 directly, and we assume that $\pi_2 = \pi_3$, i.e., that differential expression in either direction is equally likely. Thus we estimate both π_2 and π_3 as $(1 - \pi_0 - \pi_1)/2$. The two-groups model also gives estimates for μ_1 and σ_1^2, and we assign $\mu_0 = 0$ and $\sigma_0^2 = 1 \times 10^{-7}$, requiring virtually all emissions from state 0 to be 0. Finally, we estimate μ_2, σ_2^2, μ_3 and σ_2^3 with a data-driven method. We will describe the procedure for estimating μ_2 and σ_2^2; the method for μ_3 and σ_3^2 is similar.

Define n to be the total number of nonzero t-statistics that were generated from differential expression tests. Define the function n.above(x) as the observed number of nonzero t-statistics greater than x. Also define the function $c(p) = \hat{\sigma}_e \Phi^{-1}(p) + \mu_1$, where Φ represents the cumulative distribution function of the standard normal distribution. Note that for $p \in [0, 1]$, $c(p)$ yields the $100p^{th}$ percentile of the normal distribution for the equally expressed t-statistics. Using an iterative procedure, and using our estimate for π_0, we find the value $p \in [0, 1]$ such that

$$\text{n.above}[c(p)] - (1 - p)\pi_0 n = 0.25(1 - \pi_0)n \tag{1.2}$$

The reason behind finding this p is as follows: note that $0.25(1 - \pi_0)n$ is the estimate of half the number of nonzero t-statistics corresponding to bases with $D(l) = 2$: $(1 - \pi_0)n$ is the estimated number of differentially expressed bases ($D(l) = 2$ or 3), half of those have $D(l) = 2$, and we multiply by 0.5 again to get half that quantity. Also note that $(1 - p)\pi_0 n$ gives the expected number of equally expressed t-statistics greater than $c(p)$. Thus, the difference between the number of *observed* t-statistics greater than $c(p)$ and $(1 - p)\pi_0 n$ should yield the number of t-statistics *with* $D(l) = 2$ that are greater than $c(p)$. When we find a p such that this difference equals half the estimated number of t-statistics with $D(l) = 2$, we can use $c(p)$ as an estimate for the median of the distribution of overexpressed t-statistics. Since we assume this distribution is normal, $c(p)$ also provides an estimate for its mean, μ_2.

We can use μ_2 to estimate σ_2^2: assume that p solves 1.2 above, and choose any value p' in $(p, 1)$. Define the quantity:

$$q = 1 - \frac{\text{n.above}[c(p')] - (1-p')\pi_0 n}{(1-\pi_0)0.5n} \tag{1.3}$$

The numerator of the fraction in 1.3 gives the estimated number of overexpressed t-statistics greater than $c(p')$, and the denominator gives the estimated total number of t-statistics with $D(l) = 2$. Therefore, q denotes what percentile of of the distribution of $s(l) \mid D(l) = 2$ is given by $c(p')$. Then, since we know $\Phi^{-1}(q)$, $c(p')$, and μ_2, we can solve the equation

$$\Phi^{-1}(q) = \frac{c(p') - \mu_2}{\sigma_2} \tag{1.4}$$

for the unknown σ_2. We then estimate μ_3 and σ_3^2 analagously.

Numerical failure can occur in estimating μ_2, μ_3, σ_2^2, and/or σ_3^2. As backup, we estimate μ_2 with the 95th percentile of a normal distribution with mean μ_1 and variance σ_1^2, μ_3 with the 5th percentile of that distribution, and σ_2^2 and σ_3^2 with whatever was estimated for σ_1^2.

Simulation studies comparing our data-driven method to an EM algorithm, implemented with the *mclust* package (Fraley and Raftery 2002), suggest that this algorithm is more conservative (i.e., distributions for $s(l) \mid D(l) = 2$ and $s(l) \mid D(l) = 3$ are estimated to be further from the distribution of $s(l) \mid D(l) = 1$) and more computationally efficient than the EM algorithm.

Using all these pre-set and estimated parameters, the HMM is fit in *derFinder* using a Viterbi algorithm (Forney Jr 1973). In *derfinder*, the `dthmm` and `Viterbi` functions from the *Hidden-Markov* package are utilized (Harte 2012). By default, a non-stationary, homogenous HMM is fit (non-stationarity is the default in `dthmm`), though the user may fit a stationary HMM for improved computational efficiency. The model outputs the most likely state for each base-pair in the genome given the observed t-statistics.

Runtime. We suggest running DER Finder's statistical analysis (beginning with the coverage matrix as input) for each chromosome separately, since this enables the pipeline to be parallelized.

The Y chromosome presented in this manuscript took about 1 hour to run. Larger chromosomes take longer: statistical analyses of chromosomes 1 and 12 took about 27 hours and 8 hours, respectively, when the HMMs were fit as non-stationary models. Assuming stationarity had very little effect on runtime. Efforts to make the *derfinder* software more efficient are ongoing - so future releases may have improved runtimes.

Correction to previous section (Supplement, Section 1)

In the original supplement, section 1 (above), imprecise notation was used in the description of the method for estimating μ_2, σ_2^2, μ_3, and σ_3^2. Beginning at the paragraph immediately preceding equation (1.2), the supplementary material should read as follows:

Define n to be the total number of nonzero t-statistics that were generated from differential expression tests. The two-groups model is only run on these n t-statistics, which means that it gives a direct estimate of what we will call π_{0nz}, i.e., the percentage of nonzero t-statistics with true state $D(l) = 1$. Then π_1 is estimated as $\pi_{0nz}(1 - \hat{\pi_0})$, where $\hat{\pi_0}$ is the empirical estimate of the percentage of t-statistics equal to 0).

Next, define the function n.above(x) as the observed number of nonzero t-statistics greater than x. Also define the function $c(p) = \hat{\sigma}_1\Phi^{-1}(p) + \mu_1$, where Φ represents the cumulative distribution function of the standard normal distribution. Note that for $p \in [0, 1]$, $c(p)$ yields the $100p^{th}$ percentile of the normal distribution for the equally expressed t-statistics, i..e, t-statistics emitted from bases with hidden state $D(l) = 1$. Using an iterative procedure, and using our estimate for π_{0nz}, we find the value $p \in [0, 1]$ such that

$$\text{n.above}[c(p)] - (1 - p)\pi_{0nz}n = 0.25(1 - \pi_{0nz})n \qquad (1.5)$$

The reason behind finding this p is as follows: note that $0.25(1 - \pi_{0nz})n$ is the estimate of half the number of nonzero t-statistics corresponding to bases with $D(l) = 2$: $(1 - \pi_{0nz})n$

is the estimated number of differentially expressed bases ($D(l) = 2$ or 3), half of those have $D(l) = 2$, and we multiply by 0.5 again to get half that quantity. Also note that $(1 - p)\pi_{0nz}n$ gives the expected number of equally expressed t-statistics ($D(l) = 1$) greater than $c(p)$. Thus, the difference between the number of *observed* t-statistics greater than $c(p)$ and $(1 - p)\pi_{0nz}n$ should yield the number of t-statistics *with* $D(l) = 2$ that are greater than $c(p)$. When we find a p such that this difference equals half the estimated number of t-statistics with $D(l) = 2$, we can use $c(p)$ as an estimate for the median of the distribution of overexpressed t-statistics. Since we assume this distribution is normal, $c(p)$ also provides an estimate for its mean, μ_2.

We can use μ_2 to estimate σ_2^2: assume that p solves 1.5 above, and choose any value p' in $(p, 1)$. Define the quantity:

$$q = 1 - \frac{\text{n.above}[c(p')] - (1 - p')\pi_{0nz}n}{(1 - \pi_{0nz})0.5n} \tag{1.6}$$

The numerator of the fraction in 1.6 gives the estimated number of overexpressed t-statistics greater than $c(p')$, and the denominator gives the estimated total number of t-statistics with $D(l) = 2$. Therefore, q denotes what percentile of of the distribution of $s(l) \mid D(l) = 2$ is given by $c(p')$. Then, since we know $\Phi^{-1}(q)$, $c(p')$, and μ_2, we can solve the equation

$$\Phi^{-1}(q) = \frac{c(p') - \mu_2}{\sigma_2} \tag{1.7}$$

for the unknown σ_2. We then estimate μ_3 and σ_3^2 analagously.

These changes do not affect the results presented in the manuscript, but they clarify the details of the methods implemented in the manuscripts analysis and in the beta version of the DER Finder R package.

2. HMM Assumptions

Here we provide explanations and empirical evidence regarding the assumptions made in the HMM step in the DER Finder pipeline.

2.1 *Correlation*

DER Finder by default fits a first-order Hidden Markov Model. The data used as input to DER Finder is the base-by-sample coverage matrix. We expect adjacent bases to have high correlation in their coverage values, especially considering the 101-bp read length used in the Y-chromosome experiment presented. To explore the autocorrelation in coverage values across the genome, we estimated the average correlation between base-pairs at increasing distances from each other (Supplementary Figure 3). The plot does display high correlations between bases that are close together, but also shows that this correlation is close to what would be expected under an autoregressive (order 1) model. This correlation structure is acceptable under a first-order Markov model, so we believe the first-order model is sufficient for this analysis.

2.2 *Stationarity and Homogeneity*

By default, we assume that D(l) is a non-stationary, homogeneous Markov chain with hidden state probabilities $\pi_d = Pr(D(l) = d)$. However, the user may assume stationarity (i.e., constant transition and probabilities across the genome) to improve computation time, i.e., he or she may assume the transition probabilities are the same across the genome. Assuming homogeneity means we assume the parameters of the mixture distribution generating the test statistics are the same across the genome. Both these assumptions seem reasonable: even though gene density differs across the genome, the probability of staying in the same state is quite high even in areas of high gene density (i.e., transitions between states are relatively rare), since genomic features like exons and introns tend to be hundreds of bases long, but only the two bases at the beginning and end of the feature will actually show the state changing. Therefore, fitting a stationary HMM is a reasonable way to speed up computation time (though the non-stationary model can also be fit for analyses where it is computationally feasible, such as the one presented in the manuscript). Also, along the entire genome, a test statistic high in absolute value should indicate differential

expression, while a test statistic low in absolute value indicates no differential expression, so using the same parameters for the test statistics' mixture distribution seems reasonable. If the user is particularly concerned about violations of these assumptions, separate HMMs (with different parameters and transition probabilities) can be fit on different sections of the genome. A non-stationary, non-homogenous HMM could also be implemented, but functions for this may not be available off the shelf, and computation time is likely to be greatly increased. Another option is to implement an alternative segmentation algorithm, such as circular binary segmentation (Olshen *and others* 2004). This segmentation option is available in the region-finding function (called `getRegions`) in the *derfinder* R package.

2.3 *Test Statistic Distribution*

We assume that the test statistic at base l, $s(l)$ has latent state $D(l) = 1$, 2, or 3, and is a draw from a normal distribution, i.e., $s(l) \mid D(l) = d \sim N(\mu_d, \sigma_d^2)$. We choose this normal distribution because the pre-built functions in the *HiddenMarkov* R package (Harte 2012) provided the computational framework for fitting this HMM, and because the observed distribution of test statistics seemed well-captured by a normal mixture distribution. As empirical evidence, we consider the test statistics obtained from the Y chromosome analysis presented in the paper (Supplementary Figure 4): the normal mixture distribution estimated using the process described in section 1 seems to fit the observed data quite well, though the distribution of the underexpressed statistics overlaps almost entirely with that of the equally expressed statistics, which is to be expected in Y chromosome data. We also investigated the effect of the prior estimate for π_1, the proportion of base-pairs that are not differentially expressed, using the simulated data described in Section 7.1: there, we set 90% of transcripts to be differentially expressed, and DER Finder produced exactly the same results using $\hat{\pi}_1 = 0.8$, $\hat{\pi_1} = 0.9$, and $\hat{\pi_1} = 0.98$, the latter being the conservative estimate from the two-groups model. In general, we expect DER Finder

to be quite robust to choice of parameters for the test statistic distribution: as long as large test statistics are classified as differentially expressed and test statistics close to zero are classified as not differentially expressed in a systematic manner, DER Finder will produce reasonable results.

3. Validity of p-value and FDR estimates

DER Finder assignes a measure of statistical significance to each candidate DER using a permutation p-value, as described in Section 3.3 in the main text. Each candidate DER is assigned a test statistic, defined as the mean base-level statistic over all bases contained in the region. To estimate the null distribution of region-level test statistics, permutation is used, and the null distribution is created by pooling null statistics from the entire genome. Using permutation with pooled null statistics is standard practice, first introduced in Storey and Tibshirani (2003). It has been demonstrated that strong control of the FDR and FWER are guaranteed when a subset pivotality condition holds (see, e.g., Dudoit and Van Der Laan 2008). The subset pivotality requires that for any subset of the null hypotheses, the joint distribution of the p-values for the subset is identical to that under the complete null (Westfall *and others* 1993). This condition holds provided that the p-values under the null hypothesis are jointly uniform (see e.g. Leek and Storey 2011). Further justification for our approach is that this type of permutation procedure has been thoroughly studied both empirically and theoretically and is widely applied in the analysis of fMRI data: see for example, Genovese *and others* (2002) or Nichols and Holmes (2002).

We show empirically that our null p-values are uniformly distributed and that our estimated FDRs are conservative: using the simulated dataset described in Section 6.1, we analyzed all p-values from regions known to contain no differentially expressed bases and found that the distribution was approximately uniform (Supplementary Figure 5). Additionally, the true FDR in the simulation study at a q-value cutoff of 0.05 was 0, meaning our FDR estimate of 0.05 was indeed conservative. A false discovery in this case would be defined as calling a region differentially

expressed when it did not overlap a transcript set to be differentially expressed.

We can also use the Y-chromosome experiment to show that the p-values and FDR adjustments used by DER Finder's permutation test are reasonable: for the Y-chromosome data, p-value histograms for each method were created (Supplementary Figure 6). P-values were assigned to each region assigned latent state $D = 2$ by the HMM step in DER Finder, to each transcript in Cufflinks, and to each exon in EdgeR and DESeq. The observed distributions were shaped as expected in the results from DER Finder, EdgeR, and DESeq: in the comparison between sexes, many low p-values were observed, corresponding to the fact that most of the Y chromosome should be differentially expressed. However, based on the p-value histogram generated from the Cufflinks transcripts, the analysis of differential expression between sexes did not produce a very substantial number of small p-values. Instead, it produced a cluster of p-values between 0.2 and 0.4, which is an unexpected finding given the nature of Y chromosome expression differences between males and females. This simple analysis shows that the statistical methodologies used by DER Finder, EdgeR, and DESeq produce reasonable results on an easy problem, while Cuffdiff exhibits problems even in a very simple scenario.

4. Details for Y chromosome experiment

The results section of the main text presents an experiment in which we compared male and female gene expression on the Y chromosome. The data consisted of unpaired, 101-bp RNA-seq reads from 15 control samples (9 male, 6 female) of postmortem brain tissue. These reads were aligned to the Ensembl GRCh37 genome (Illumina 2012) using Tophat version 2.0.8 with default parameters, which allow mutiple alignments per read to be reported. DER Finder's coverage matrix was calculated based on these Tophat alignments. Results from DER Finder were compared to results from the Cufflinks/Cuffdiff pipeline, EdgeR, and DESeq. EdgeR and DESeq analyses were run at the exon level, using exon-by-sample count tables created based on the Tophat alignment

file with RSamtools (Morgan and Pagès) and GenomicRanges (Aboyoun *and others*). Exon-level expression summaries for EdgeR and DESeq were calculated using the `summarizeOverlaps` function in GenomicRanges, using the union model to count reads falling within overlapping exons (`mode="Union"` option in `summarizeOverlaps`). Default parameters and library size adjustments were used in EdgeR and DESeq. For these exon-by-sample count tables and for determining DER Finder's regions' overlaps with exons, we considered all annotated exons in the Ensembl GRCh37 build, as annotated in the databases used by the biomaRt Bioconductor package (Durinck *and others* 2005).

For Cufflinks/Cuffdiff, we used Cufflinks version 2.0.2 for transcript assembly and Cuffdiff version 2.0.2 for differential expression analysis. Default parameters were used for both steps. Detailed commands used are available upon request from the corresponding authors.

The main model for DER Finder (model 3.1) was fit as follows: g was defined as the function $g(x) = log_2(x + 32)$, $X_{2i} = 1$ if sample i was male and 0 if sample i was female (this is essentially the case/control scenario, so $P = 2$), and W_{i1} was defined as the median of nonzero coverage values for each sample. Using this model setup, $\hat{\beta}_2(l_j)$ represents the estimated log (base 2) fold change in expression of base l_j for males compared to females, when all coverage values are offset by 32 to ensure that zero counts would not cause problems in the log transformation. No other confounders were included in the analysis. The test statstic on the base level was *limma*'s moderated t statistic (see section 1), and the HMM with *derfinder*'s default parameters was run on these t statistics to obtain candidate DERs (details are described in the supplement). To obtain p-values for the candidate DERs, a permutation test was run as described in section 3.3 of the main manuscript, using $B = 10$ permutations. All p-values (from all pipelines) were adjusted for multiple testing by controlling the false discovery rate, so the q-value (Storey and Tibshirani 2003) was used as a measure of statistical significance.

To connect the results from this experiment to annotated features, we labeled each DER

with what type of genomic event it might indicate and which annotated features are involved (Supplementary Table 1). These labels aid in determining which exons and genes are showing differential expression signal and finding regions that may indicate phenomena like possible alternative splicing. Further exploration of these regions is possible using assemble-then-annotate methods to evaluate potential alternative or differential splicing events. Due to variance in read coverage across the genome, we observed some regions shorter than the length of an individual read. These small regions are particularly detrimental in the annotation and labeling step. We therefore choose to disregard regions shorter than the read length. Regions flanking very short transitions between states are merged.

5. Additional figures illustrating problems with annotate-then-identify methods

Figure 5 in the main text illustrates specific instances in the analysis of the human Y chromosome where DER Finder correctly identifies differential expression between sexes and EdgeR and DESeq do not, either because an exon was incorrectly annotated or because the differential expression did not occur within an exon at all. The instances shown in the text are not isolated: in fact, 280 non-exonic regions of the Y chromosome were identified by DER Finder as significantly differentially expressed ($q < 0.05$).

Additionally, Supplementary Figure 1 demonstrates that differential expression does not always occur within exon boundaries, and as such, an identify-then-annotate method may be necessary to achieve high sensitivity. We examined differentially expressed Y-chromosome regions found by DER Finder that overlapped only part of an exon: for a fixed percentage x, we gathered the DERs (identified with DER Finder) that overlapped no more than $x\%$ of an exon. Then we calculated the fraction of the set exons overlapped by those DERs that EdgeR and DESeq called differentially expressed ($q < 0.05$). Supplementary Figure 1 plots different values of x against

these fractions. The figure's message is that many exons showing a differential expression signal when analyzed with DER Finder are not called differentially expressed by EdgeR and DESeq, even in an easy analysis of differential expression between males and females on the Y chromosome. Figure 2 in the main text is a specific example of this problem, and Supplementary Figure 1 suggests that the issue is not confined to only one example.

Supplementary Figure 2 shows how DER Finder's agreement with EdgeR and DESeq's findings changes based on how much of an exon we require a DER to overlap in order to call that exon differentially expressed. (While Supplementary Figure 1 looked at how much EdgeR and DESeq agreed with DER Finder's results, this figure examines how much DER Finder agrees with EdgeR and DESeq's results). Given a percentage x to use as a cutoff for how much an exon must be overlapped by a DER in order to be called differentially expressed by DER Finder, Supplementary Figure 2 shows how many of the exons identified by DESeq or EdgeR as differentially expressed are also $x\%$ covered by a DER. As more overlap is required for a differential expression call, the percent agreement between DER Finder and the identify-then-annotate methods decreases, but overall, most of the exons identified by EdgeR and DESeq also show a signal in DER Finder.

Together, these two figures show that DER Finder identifies most of the differential expression found by EdgeR and DESeq, but the identify-then-annotate methods miss signals identified by DER Finder due to the heavy reliance on pre-specified exon annotation.

6. Additional Y-chromosome analysis: agreement between methods

To determine the extent to which the different pipelines discovered the same features to be differentially expressed, we quantified differential expression and overlap between findings at varying q-value cutoffs, comparing DER Finder to Tophat-Cufflinks-Cuffdiff (Supplementary Table 2) and DER Finder to EdgeR and DESeq (Supplementary Table 3). The new method produces better results than Cufflinks: we find differential expression between males and females on the Y

chromosome, and find no differential expression between the males, while Cufflinks does not find differential expression between sexes unless the q-value cutoff is above 0.45. When the q-value cutoff is high (0.50), only 5.3% of the differentially expressed Cufflinks transcripts are also called differentially expressed by the new method: as expected, transcripts with high q-values are not overlapped by differentially expressed regions from the new method (regions that are equally expressed will not make it past the HMM segmentation step). On the other hand, 32.5% of the differentially expressed regions ($q < 0.50$) are overlapped by differentially expressed transcripts, which shows some agreement between the methods.

The comparison to EdgeR and DESeq shows the annotation-based results to be somewhat similar. The q-value cutoff did not seem to matter when assessing exon-specific results from DER Finder for the male-to-female comparison of the Y chromosome (Supplementary Table 3). Overall, the DER Finder results and the EdgeR and DESeq results were somewhat comparable on the exon level. The q-value cutoff had no bearing on the DER Finder results: all the differentially expressed regions covering at least 80% of an annotated exon had small q-values. At low q-values, DER Finder identifies more exons as differentially expressed than EdgeR and DESeq do, with some agreement between all three methods. Results from Supplementary Table 3 are from the comparison between sexes; the males showed no differential exon expression in EdgeR/DESeq (all but two q-values 1), or DER Finder (minimum q-value of 0.86). It is worth noting that the method of summarizing the number of reads per exon affects EdgeR and DESeq results: in particular, the common counting methods do not allow reads to be counted toward more than one feature, so overlapping exons do not usually get any reads assigned to them at all. In our experiment, this led to 345 exons having overlapping DERs assigned to them but not even being tested by EdgeR or DESeq. This issue explains some of the discrepancy between the exon-level findings for DER Finder and EdgeR/DESeq. Also, though DER Finder identifies more exons overall as being differentially expressed, 54 exons are identified only by EdgeR or DESeq.

Closer examination of the coverage patterns of these exons revealed that most of them were either (a) very lowly expressed overall, or (b) were less than 80% covered by DERs, so the exons themselves were not called differentially expressed because of the cutoffs defined in Table 1 of the main manuscript. Users can adjust DER Finder parameters if they are particularly interested in discovering differential expression of lowly-expressed features (e.g., the function $g()$ chosen in model 3.1 could be $g(x) = log_2(x + 0.5)$ rather than $log_2(x + 32)$, which is what was was used the Y chromosome comparison). Also, DER Finder generally does show *signal* in the general area of the exons in question, even if that signal does not overlap the exon by 80%, so the results still give meaningful information. Overall, these findings confirm the result that EdgeR, DESeq, and DER Finder perform similarly when analyzing already-annotated features.

7. Experimental Design Concerns

Biologists who collect RNA-seq data must make several decisions when designing their experiments. Two important considerations are whether to use single-end or paired-end reads and how deeply to sequence the samples. We address these considerations and their impact on DER Finder's results in this section, using a small simulation study to support the conclusions drawn.

7.1 *Simulation set-up*

A small, 20-sample RNA-seq dataset with pre-defined differential expression was simulated using Flux Simulator version 1.2 (Griebel *and others* 2012). We simulated 76-bp paired-end reads from 1000 randomly selected transcripts on chromosome 22. For these 1000 transcripts, we simulated approximately 400,000 reads per sample. We then randomly chose 50 of these transcripts to be overexpressed in 10 of the samples (group A) and 50 different transcripts to be overexpressed in the other 10 samples (group B). Overexpression was simulated by generating an additional 80,000 reads from the designated 50 transcripts for each sample. Essentially, this process mimicked

a 5x fold change per overexpressed transcript. The default error model for 76-bp reads was utllized, and all other parameters were left at the default value. The command run for each simulated sample was `flux-simulator -t simulator -x -l -s -p sample.par`. An example parameter (.par) file is available on github. The simulated reads from each dataset were aligned to the Ensembl GRCh37 genome (Illumina 2012) using Tophat 2.0.8 with default parameters, and coverage matrices were created from the Tophat alignment file.

7.2 *Paired-end data in RNA-seq analysis*

It is generally accepted that using paired-end data, i.e., data consisting of reads from both ends of the mRNA fragments instead of just one end, is better than using single-end data, even though paired RNA-seq experiments can cost up to twice as much as a single-end experiment (Katz *and others* 2010, Trapnell *and others* 2012). Mate-pair information is used during read alignment to more accurately determine the reads' best mappings. Furthermore, paired-end data is especially important in assemble-then-identify methods because it yields more reliable transcript assemblies and better per-transcript abundance estimates. Because annotate-then-identify and identify-then-annotate methods do not involve assembly or transcript-level quantification, paired-end data only improves these methods inasmuch as it improves the read alignment step. Therefore, since read alignment can be done with either single-end or paired-end reads, it is appropriate to use DER Finder with either type of data. The coverage matrix would be calculated the same way for paired data as it is for single-end data; each mate of a mate pair would contribute a coverage value of 1 to all the bases to which it aligns.

The Y-chromosome analysis in this manuscript was done using single-end data, which may put Cufflinks/Cuffdiff (the assemble-then-identify method) at a disadvantage when comparing it to the other tools. To determine whether the poor statistical results from Cufflinks/Cuffdiff may have been due to using single-end data, we ran Cufflinks and Cuffdiff (version 2.0.2, with

default parameters) on the simulated dataset. Even though this dataset was paired-end and contained transcripts known to be highly differentially expressed, the statistical results from Cufflinks/Cuffdiff were unreasonable: they did not reflect any differential expression (Supplementary Figure 7). Therefore, we contend that while paired-end data may improve assembly methods, it is not the deciding factor in whether the Cufflinks/Cuffdiff pipeline produces reasonable statistical results.

7.3 *Effect of sequencing depth*

Sequencing depth (or read coverage) refers to how many times each mRNA nucleotide in the sample is read by the sequencing machine. Experiments with greater sequencing depth are better able to detect expression differences for features that are lowly expressed overall. This property holds for most existing differential expression analysis methods, including DER Finder. Therefore, experimenters wishing to use DER Finder and detect differential expression in for lowly expressed feature should deeply sequence their samples. One specific consideration in DER Finder is the choice of $g()$ in model (3.1). In general, we recommend using $g(x) = log_2(x + k)$, where k is a constant that allows the method to avoid taking the log of 0. In our experiment, we set $k = 32$ because we were not particularly interested in differential expression in areas with low coverage, and offsetting all counts by 32 attenuates the fold changes observed in very low-coverage regions. However, if the sequencing depth is high, the user may want to increase k (if lowly-expressed features are not of interest), since the method will be more sensitive to differential expression of lowly-expressed features with deep sequencing. Similarly, if the samples are not sequenced very deeply, the user may want to decrease k, since true differential expression may not be detected if the samples' coverage values are offset too much.

We also investigated the effect of sequencing depth using the simulated dataset described above in addition to two more simulated datasets. These additional datasets were generated

in the same manner as the first dataset except for read coverage: the first additional dataset had half as many reads as the original dataset, and the second had 1/4 as many reads as the original dataset. Based on the median length of the transcripts included in these experiments, the coverages for these datasets were approximately 24x, 12x, and 6x, respectively. Coverage matrices were created using Tophat alignments, and DER Finder was run on the chromosome 22 coverage matrix for each dataset, with model 3.1 defined as follows: $g(x) = log_2(x + 32)$, $X_i = 1$ for samples in group A and 0 for samples in group B, and W_{i1} was set as the median nonzero coverage value for each sample.

In this simulated dataset, DER Finder using the 24x and 12x datasets found 435 and 433 differentially expressed regions, respectively ($q < 0.05$), while the 6x dataset did not find any differential expression (minimum q-value 0.18). This is consistent with what we expect: the same offset ($k = 32$) was used for all three datasets, and this appears to be too much of an offset for the low-coverage (6x) dataset. To further investigate these findings, we used varying q-value cutoffs to create ROC curves for the different coverage levels (Supplementary Figure 8). DER Finder appears to be performing well in terms of sensitivity and specificy for the 12x and 24x experiments: for example, in the 24x experiment, 97 out of the 100 pre-set differentially expressed transcripts were overlapped by a significant ($q < 0.05$) DER, while 93% of the transcript features (exons, etc.) that were not simulated as differentially expressed were overlapped by regions in the equally expressed state or with $q \geqslant 0.05$. In general, there was very little difference between 12x and 24x coverage in this simulation, but 6x read coverage appears to be too shallow when the offset is set at 32.

8. Code

Code - both general R functions and code used to do this particuar analysis - is available on github: (https://github.com/alyssafrazee/derfinder).

References

Aboyoun, P., Pages, H. and Lawrence, M. *GenomicRanges: Representation and manipulation of genomic intervals*. R package version 1.6.7.

Dudoit, Sandrine and Van Der Laan, Mark J. (2008). *Multiple testing procedures with applications to genomics*. Springer.

Durinck, Steffen, Moreau, Yves, Kasprzyk, Arek, Davis, Sean, De Moor, Bart, Brazma, Alvis and Huber, Wolfgang. (2005). BioMart and Bioconductor: a powerful link between biological databases and microarray data analysis. *Bioinformatics* **21**(16), 3439–3440.

Efron, B. (2008). Microarrays, empirical Bayes and the two-groups model. *Statistical Science* **23**(1), 1–22.

Forney Jr, G.D. (1973). The viterbi algorithm. *Proceedings of the IEEE* **61**(3), 268–278.

Fraley, C. and Raftery, A.E. (2002). Model-based clustering, discriminant analysis, and density estimation. *Journal of the American Statistical Association* **97**(458), 611–631.

Genovese, Christopher R, Lazar, Nicole A and Nichols, Thomas. (2002). Thresholding of statistical maps in functional neuroimaging using the false discovery rate. *Neuroimage* **15**(4), 870–878.

Griebel, T., Zacher, B., Ribeca, P., Raineri, E., Lacroix, V., Guigó, R. and Sammeth, M. (2012). Modelling and simulating generic RNA-Seq experiments with the flux simulator. *Nucleic Acids Research*.

Harte, David. (2012). *HiddenMarkov: Hidden Markov Models*. Statistics Research Associates, Wellington. R package version 1.7-0.

ILLUMINA. (2012). Illumina iGenomes. `http://cufflinks.cbcb.umd.edu/igenomes.html`.

KATZ, YARDEN, WANG, ERIC T, AIROLDI, EDOARDO M AND BURGE, CHRISTOPHER B. (2010). Analysis and design of RNA sequencing experiments for identifying isoform regulation. *Nature methods* **7**(12), 1009–1015.

LEEK, JEFFREY T AND STOREY, JOHN D. (2011). The joint null criterion for multiple hypothesis tests. *Statistical Applications in Genetics and Molecular Biology* **10**(1).

MORGAN, MARTIN AND PAGÈS, HERVÉ. *Rsamtools: Binary alignment (BAM), variant call (BCF), or tabix file import*. R package version 1.6.3.

NICHOLS, THOMAS E AND HOLMES, ANDREW P. (2002). Nonparametric permutation tests for functional neuroimaging: a primer with examples. *Human brain mapping* **15**(1), 1–25.

OLSHEN, ADAM B, VENKATRAMAN, ES, LUCITO, ROBERT AND WIGLER, MICHAEL. (2004). Circular binary segmentation for the analysis of array-based DNA copy number data. *Biostatistics* **5**(4), 557–572.

SMYTH, G.K. (2005). Limma: linear models for microarray data. In: Gentleman, R., Carey, V., Dudoit, S., Irizarry, R. and Huber, W. (editors), *Bioinformatics and Computational Biology Solutions using R and Bioconductor*. New York: Springer, pp. 397–420.

SMYTH, G.K. *and others*. (2004). Linear models and empirical bayes methods for assessing differential expression in microarray experiments. *Statistical Applications in Genetics and Molecular Biology* **3**(1), 3.

STOREY, J.D. AND TIBSHIRANI, R. (2003). Statistical significance for genomewide studies. *Proceedings of the National Academy of Sciences* **100**(16), 9440–9445.

TRAPNELL, COLE, ROBERTS, ADAM, GOFF, LOYAL, PERTEA, GEO, KIM, DAEHWAN, KELLEY, DAVID R, PIMENTEL, HAROLD, SALZBERG, STEVEN L, RINN, JOHN L AND PACHTER, LIOR.

(2012). Differential gene and transcript expression analysis of RNA-seq experiments with TopHat and Cufflinks. *Nature Protocols* **7**(3), 562–578.

Westfall, PH, Young, SS and Wright, S Paul. (1993). On adjusting p-values for multiplicity. *Biometrics* **49**(3), 941–945.

9. Figures and Tables

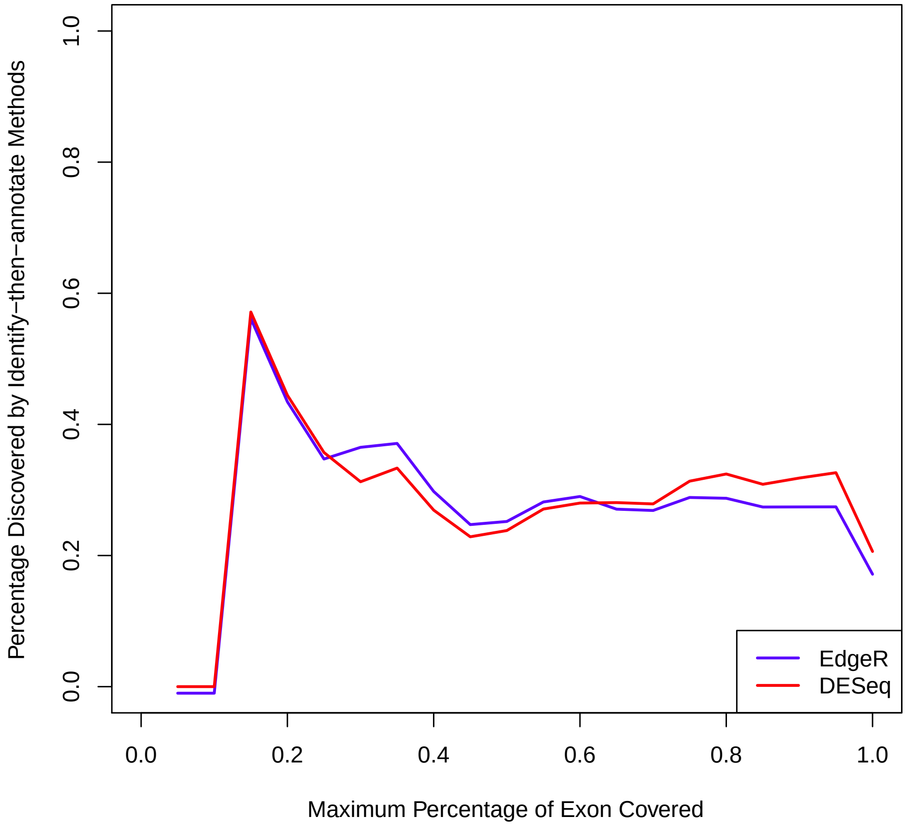

Fig. 1. Percentage of the exons overlapped by no more than $x\%$ (for varying values of x) of a differentially expressed region ($q < 0.05$) from DER Finder that are also identified as differentially expressed ($q < 0.05$) by EdgeR and DESeq. (The EdgeR line was lowered by 0.01 so the differences between the two lines on the left side of the plot would be visible.)

**Result**	**Flag**
A set of regions of state $D = 2$ overlaps more than 80% of an annotated exon.	Differentially Expressed Exon
There exists a set of regions of state $D = 2$ with differentially expressed exon flags such that all exons in a given gene are flagged by the set	Differentially Expressed Gene
There exists a set of regions of state $D = 2$ with differentially expressed exon flags such that at least one, but not all, of the exons in a given gene are flagged by the set	Unknown Event of Interest (e.g., alternative splicing)
Region of state $D = 1$ does not overlap any annotated exons	Novel Transcribed Region
Region of state $D = 2$ does not overlap any annotated exons	Novel Differentially Transcribed Region

Table 1. Genomic events indicated by HMM results

**q-value**	**# DE regions**	**# DE transcripts**	**# agreeing regions**	**# agreeing transcripts**
		(a) males vs. females		
0.05	534	0	NA	0
0.10	1009	0	NA	0
0.50	1185	758	40	385
0.80	1259	787	48	412
		(b) males vs. males		
0.05	0	0	NA	NA
0.10	0	0	NA	NA
0.50	0	0	NA	NA
0.80	0	458	0	NA

Table 2. Comparison of results from DER Finder to Tophat-Cufflinks-Cuffdiff. The first column is the number of differentially expressed regions found by DER Finder, while the second column is the number of differentially expressed transcripts found by Cufflinks, both at the specified q-value cutoff. The third column shows how many of the differentially expressed Cufflinks transcripts are at least 80% overlapped by a differentially expressed region from DER Finder, while the fourth column shows how many of the differentially expressed regions are at least 80% overlapped by a differentially expressed Cufflinks transcript.

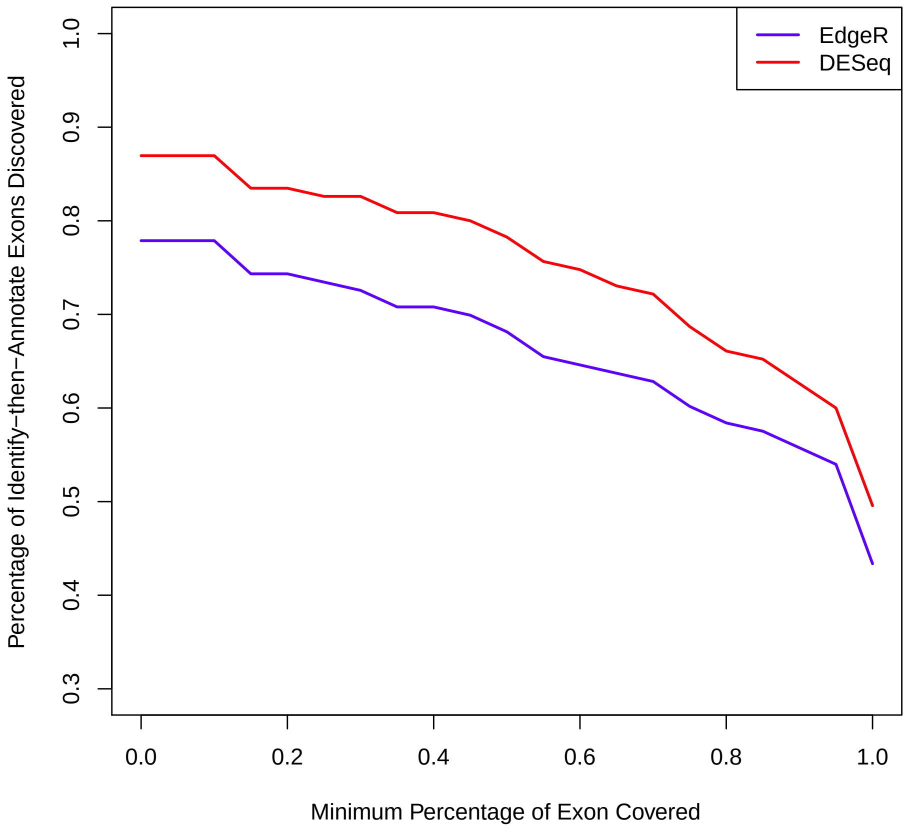

Fig. 2. Percentage of exons called differentially expressed ($q < 0.05$) by EdgeR and DESeq that are overlapped by at least x% of a differentially expressed region ($q < 0.05$) from DER Finder, for varying values of x.

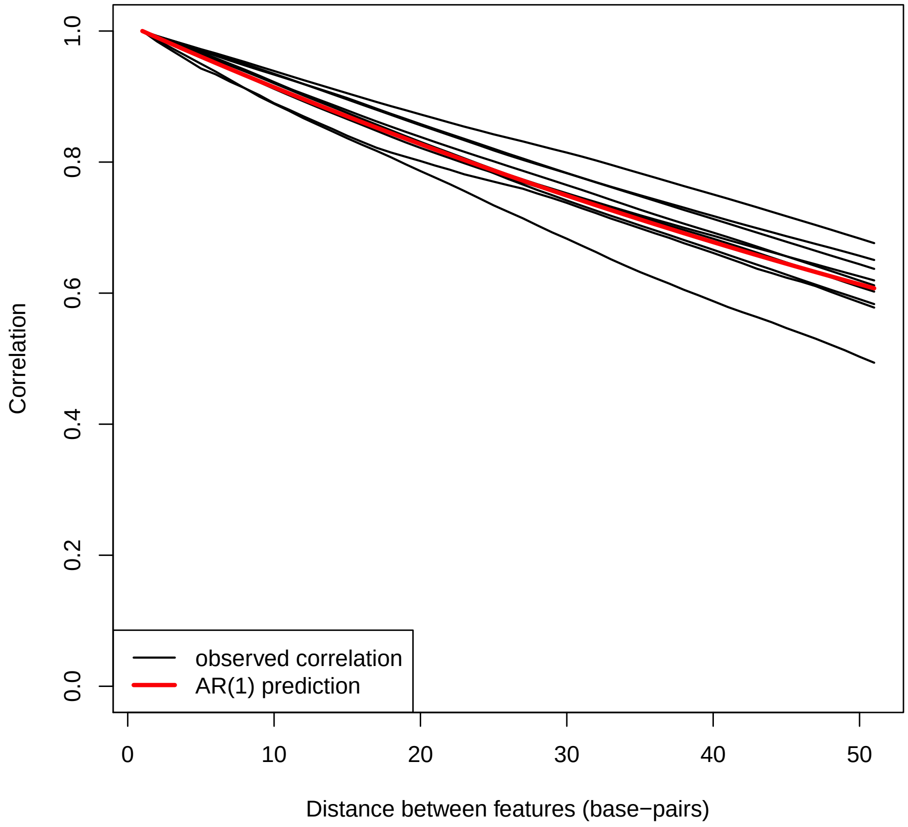

Fig. 3. Observed average correlation (y-axis) between bases of varying distances apart (x-axis), with the predicted AR(1) correlation for this data superimposed in red. Each black line represents one of the nine male samples used in the Y-chromosome analysis.

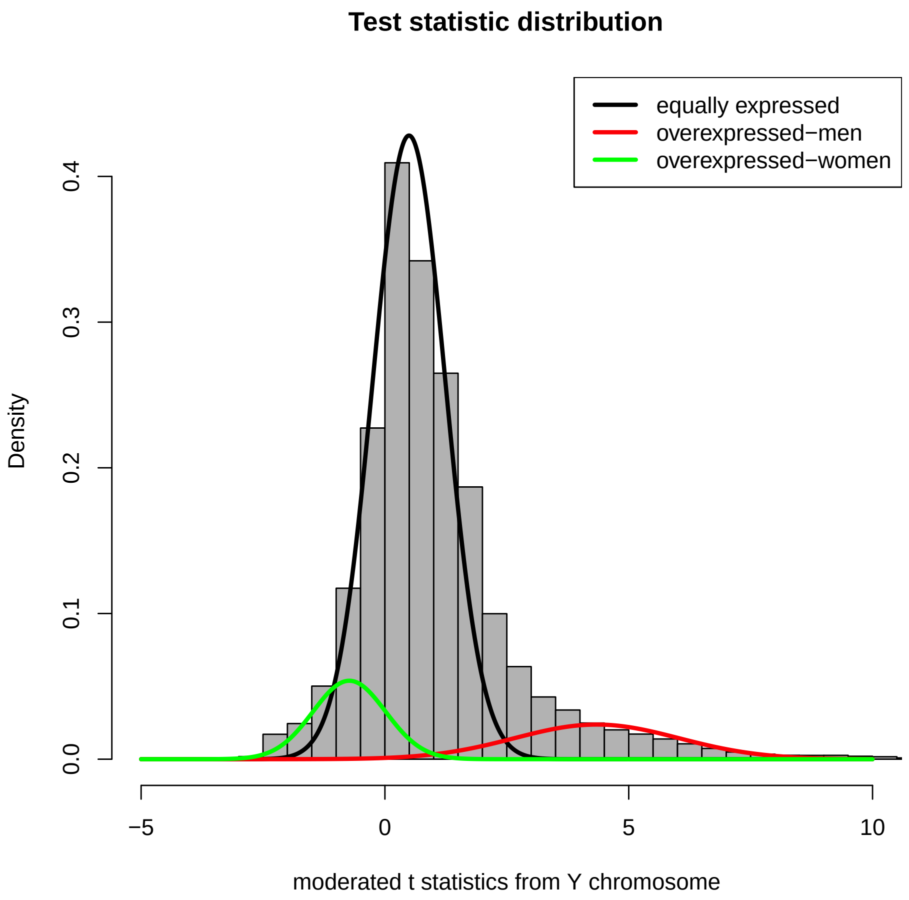

Fig. 4. Estimated normal mixture distribution of test statistics generated from bases on the Y chromosome. This figure illustrates the plausibility of the assumption that $s(l) \mid D(l) = d \sim N(\mu_d, \sigma_d^2)$. The separate components of the mixture distribution are plotted in different colors.

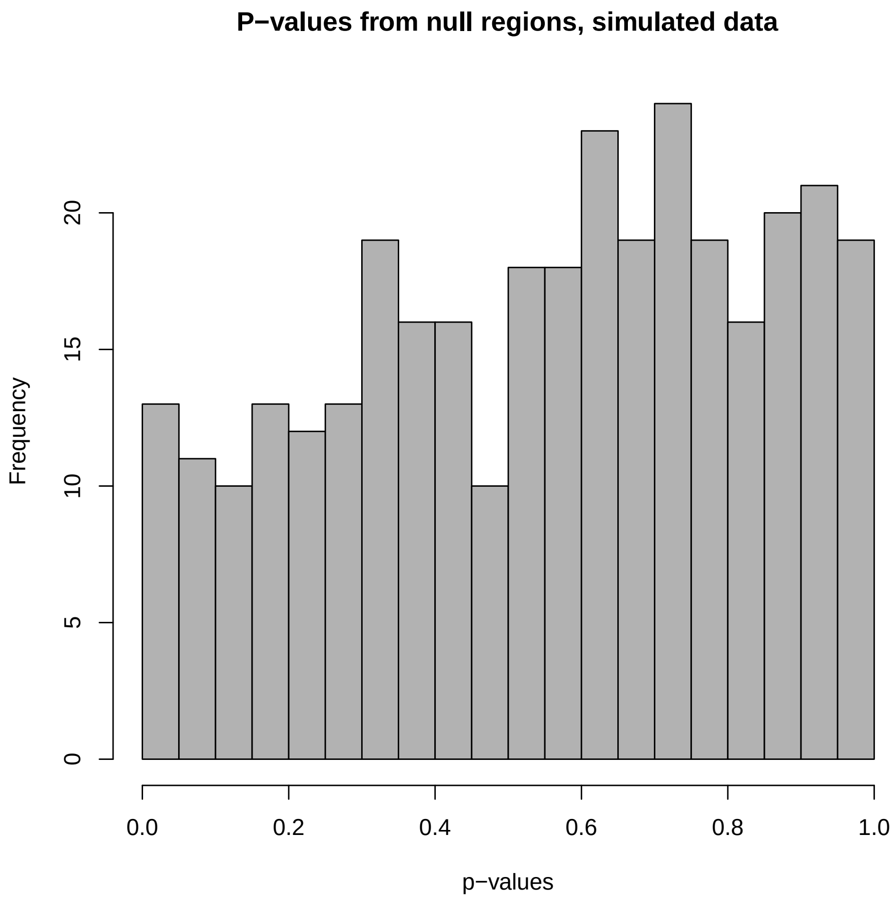

Fig. 5. Histogram of null p-values from a small simulation study, where a region is considered null if none of the bases in that region were contained in a transcript that was set to be differentially expressed. This distribution is approximately uniform, which implies that these p-values have good theoretical properties.

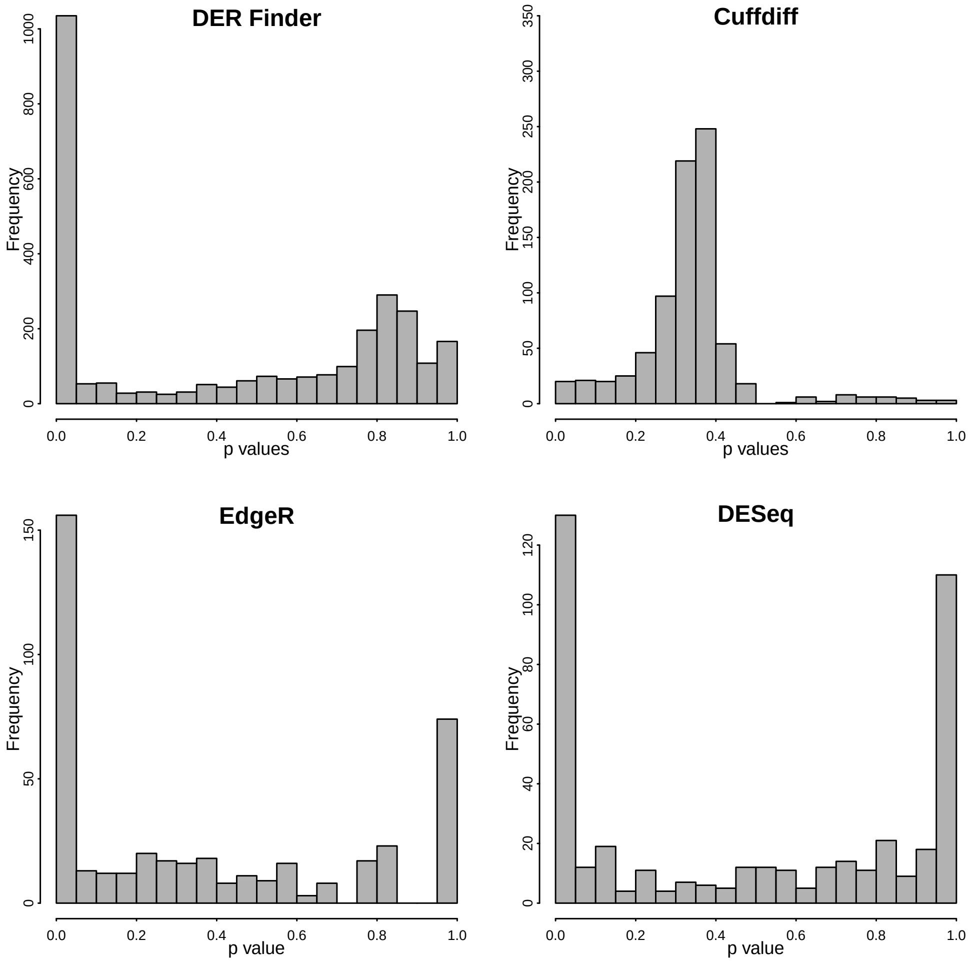

Fig. 6. P-value histograms for tests of differential expression on the Y chromosome between males and females. For all methods except Cufflinks, substantial differential expression is evident in the comparisons between sexes, as expected. The Cufflinks p-value distribution is quite unusual and indicates that using p-values adjusted for multiple testing to assess significance may be problematic.

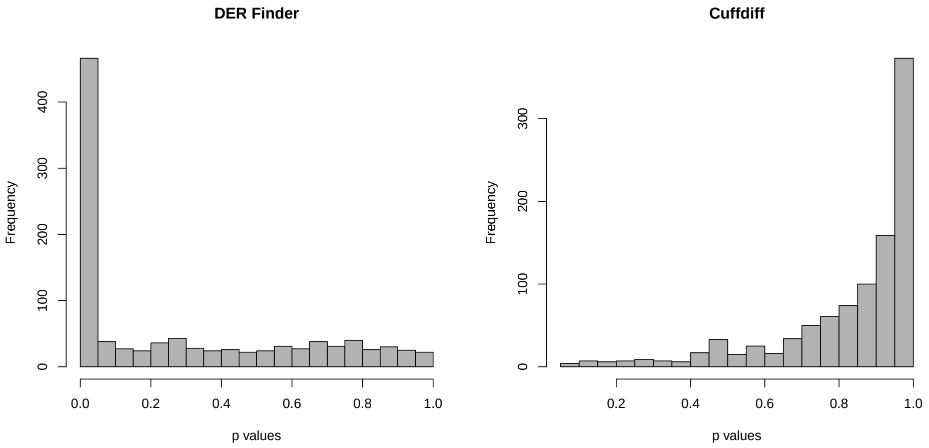

Fig. 7. P-value histograms from a small, paired-end simulation study with known differentially expressed transcripts. DER Finder's p-values have the expected distribution, while Cuffdiff produces unreasonable statistical results, calling nothing differentially expressed (minimum q-value 0.999) despite 10% of transcripts being overexpressed (fold change = 5) in one condition. This figure demonstrates that paired-end sequencing does not eliminate the problems with Cuffdiff's statistical analysis.

**q-value**	**# DE Regions**	**# DE DER Finder exons**	**# DE EdgeR exons**	**# DE DESeq exons**	**DER Finder /EdgeR overlap**	**DER Finder /DESeq overlap**	**EdgeR /DESeq overlap**	**All overlap**
0.05	534	411	113	115	66	76	97	65
0.10	1009	417	125	120	76	81	106	74
0.50	1185	417	143	165	80	86	127	79
0.80	1259	417	153	187	83	89	134	82

Table 3. Comparison of results from DER Finder to EdgeR and DESeq, analyzing differential expression at the exon level on the Y chromosome between males and females. The first column is the number of differentially expressed regions found by DER Finder, and the second, third, and fourth columns are the number of differentially expressed exons found by each method at the specified q-value cutoff. Differentially expressed exons for DER Finder were defined as exons that were more than 80% covered by regions of state $D = 2$; the q-value for each exon was taken to be the q-value of the region most overlapping it. The last four colums show the number of exons found by two or all three methods.

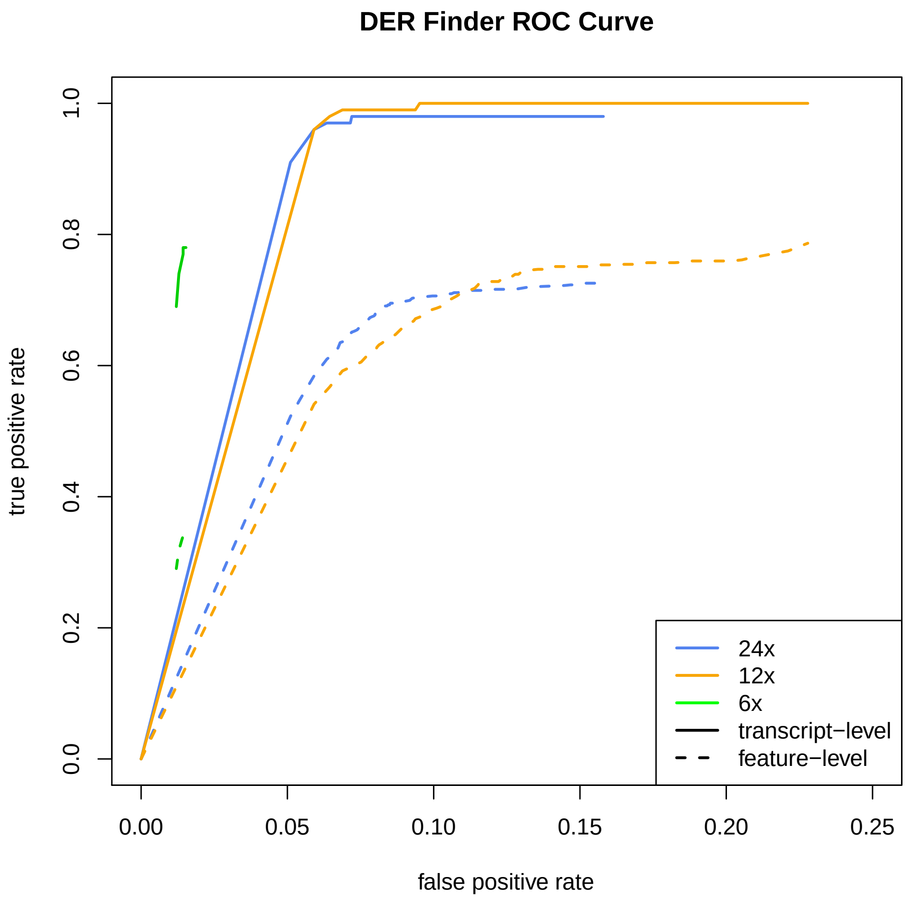

Fig. 8. ROC curves from DER Finder, created based on the simulation study with known differential expression. Sequencing depth is noted by color, while line type denotes different ways of determining differential expression calls: the dashed lines were created at the feature level, i.e., the true positive rate was the percentage of differentially expressed transcript *features* (exons, etc.) that were overlapped by a significant DER. The solid lines were created at the transcript level, i.e., the true positive rate was the percentage of *transcripts* with at least one feature overlapped by a significant DER. DER Finder is performing well in terms of sensitivity and specificity when the sequencing depth is sufficient.
