# Supplementary material for: Corrigendum: Differential expression analysis of RNA-seq data at single-base resolution
Source: Biostatistics. 2014 Jul;15(3):584–5. doi: 10.1093/biostatistics/kxu022 (PMC4059467; doi:10.1093/biostatistics/kxu022)
Supplement: Supplementary Data [file supp_15_3_584__index.html]

Corrigendum: Differential expression analysis of RNA-seq data at single-base resolution — Supplementary Data 

# Corrigendum: Differential expression analysis of RNA-seq data at single-base resolution

## Supplementary Data

Supplementary Data

**Files in this Supplementary Material:**

- Supplementary Data - Pdf file
